# Genetic Interactions Involving Five or More Genes Contribute to a Complex Trait in Yeast

**DOI:** 10.1371/journal.pgen.1004324

**Published:** 2014-05-01

**Authors:** Matthew B. Taylor, Ian M. Ehrenreich

**Affiliations:** Molecular and Computational Biology Section, Department of Biological Sciences, University of Southern California, Los Angeles, California, United States of America; Washington University School of Medicine, United States of America

## Abstract

Recent research suggests that genetic interactions involving more than two loci may influence a number of complex traits. How these ‘higher-order’ interactions arise at the genetic and molecular levels remains an open question. To provide insights into this problem, we dissected a colony morphology phenotype that segregates in a yeast cross and results from synthetic higher-order interactions. Using backcrossing and selective sequencing of progeny, we found five loci that collectively produce the trait. We fine-mapped these loci to 22 genes in total and identified a single gene at each locus that caused loss of the phenotype when deleted. Complementation tests or allele replacements provided support for functional variation in these genes, and revealed that pre-existing genetic variants and a spontaneous mutation interact to cause the trait. The causal genes have diverse functions in endocytosis (*END3*), oxidative stress response (*TRR1*), RAS-cAMP signalling (*IRA2*), and transcriptional regulation of multicellular growth (*FLO8* and *MSS11*), and for the most part have not previously been shown to exhibit functional relationships. Further efforts uncovered two additional loci that together can complement the non-causal allele of *END3*, suggesting that multiple genotypes in the cross can specify the same phenotype. Our work sheds light on the complex genetic and molecular architecture of higher-order interactions, and raises questions about the broader contribution of such interactions to heritable trait variation.

## Author Summary

Although it is well known that interactions among genetic variants contribute to many complex traits, the forms of these interactions have not been fully characterized. Most work on this problem to date has focused on relatively simple cases involving two or three loci. However, higher-order interactions involving larger numbers of loci can also occur, and may have significant effects on the relationship between genotype and phenotype. In this paper, we dissect a colony morphology trait that segregates in a cross of two yeast strains and is caused by genetic interactions among five or more loci. Our work demonstrates that higher-order interactions can have major phenotypic effects, and provides novel insights into the genetic and molecular basis of these interactions.

## Introduction

Understanding the genetic basis of complex traits is critical for advancing medicine, evolutionary biology, and agriculture [1], [2]. A challenge to progress in this area is that genetic variants can interact, resulting in unexpected phenotypic consequences [3]–[7]. Most of our knowledge about these genetic interactions in natural systems comes from studies focused on two-locus interactions where at least one of the loci exhibits a measurable effect on its own (e.g., [8]). However, evidence suggests that genetic interactions involving three or more loci also occur [9], [10], and that loci participating in such interactions may not individually have detectable effects [11]. Determining how these higher-order interactions arise and influence phenotypic variation could help solve the ‘missing heritability’ problem faced by geneticists studying humans and model species [12].

In this paper, we describe the genetic basis of a complex trait that is influenced by higher-order interactions. We identified this phenotype, a dramatic change in the morphology of *Saccharomyces cerevisiae* colonies, in a cross of haploid derivatives of the lab strain BY4716 and the clinical isolate 322134S (hereafter ‘BY’ and ‘3S’, respectively). The colony morphology trait in the BY×3S cross is similar to phenotypes described in other yeast isolates and crosses (e.g., [13]–[19]). Thus, by comprehensively determining the genetic basis of colony morphology variation among BY×3S offspring, we not only generate novel insights into how higher-order interactions contribute to phenotypic variation, but also provide new information regarding the genetic basis of a frequently studied model complex trait.

## Results and Discussion

**Figure 1:**
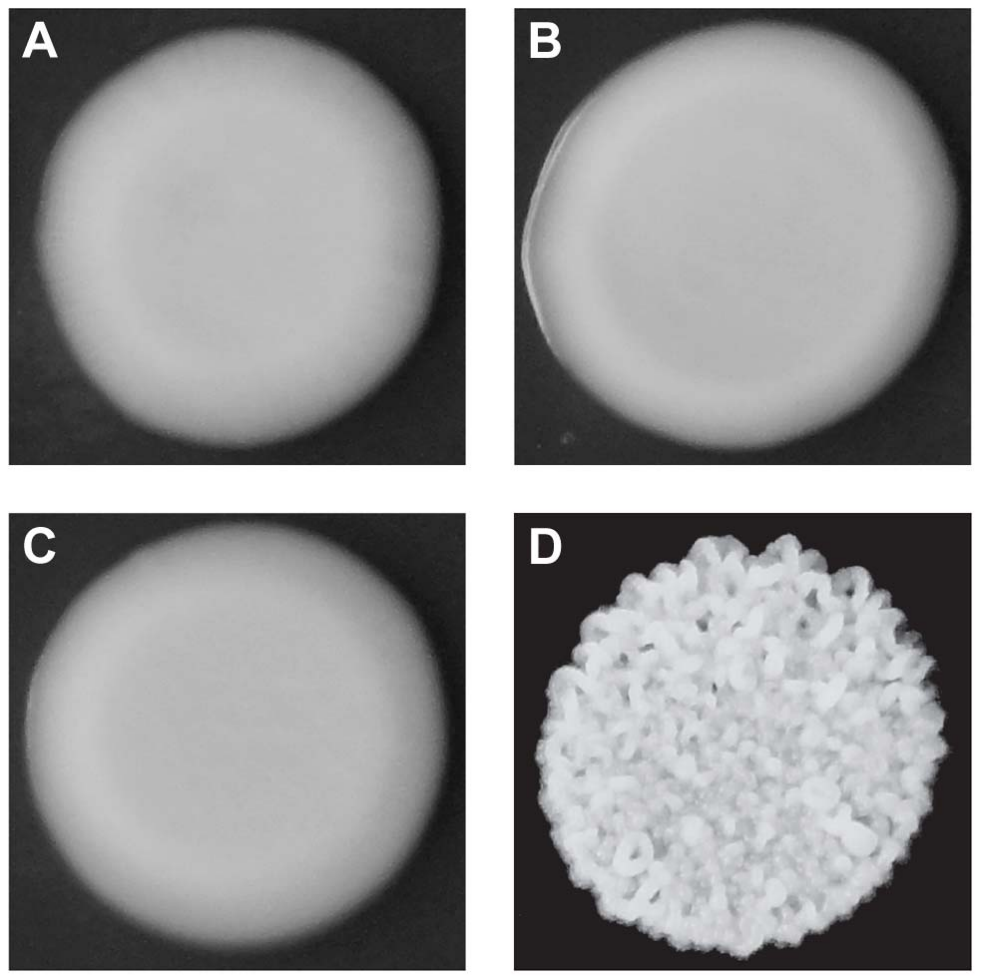
Colony morphologies of parents and cross progeny on rich medium containing ethanol. 3S (A) and BY (B) each form smooth colonies. When these two strains are crossed, most offspring also form smooth colonies, but a small fraction form rough colonies. An example smooth segregant is shown in C and an example rough segregant is shown in D.

Although both BY and 3S, as well as most of their haploid offspring, form smooth colonies (Figure 1A–C), ∼2% of their progeny exhibited rough colonies when we examined 250 segregants (Figure 1D). Previous work has shown that such heritable variation in colony morphology in *S. cerevisiae* can arise due to naturally occurring polymorphisms or spontaneous mutations at chromosomal loci [13], [14], [18], [19], aneuploidies [17], and prions [15]. Unlike chromosomal loci, which should show stable inheritance across generations, aneuploidies and prions can be gained or lost, resulting in phenotypic switching. Multiple lines of evidence suggest that chromosomal loci are the primary cause of rough morphology in the BY×3S cross. Neither BY nor 3S exhibits rough morphology, indicating that the phenotype likely requires a combination of alleles from both of these strains. Consistent with this statement, we found that the frequency of rough morphology increased to 12.5% and 21.2% among recombinant haploid progeny obtained by backcrossing a rough segregant to BY and 3S, respectively (Tables S1 and S2; Methods). The higher frequency of rough segregants in backcrosses is expected if alleles from both parents contribute to the trait, as fewer causative alleles should segregate in the backcrosses than in the original cross. Further supporting the argument that our observations of rough morphology were due to chromosomal loci instead of transient factors, we found no evidence for chromosome-scale aneuploidies or phenotypic switching in the backcrossed segregant (Figure S1; Methods).

**Figure 2:**
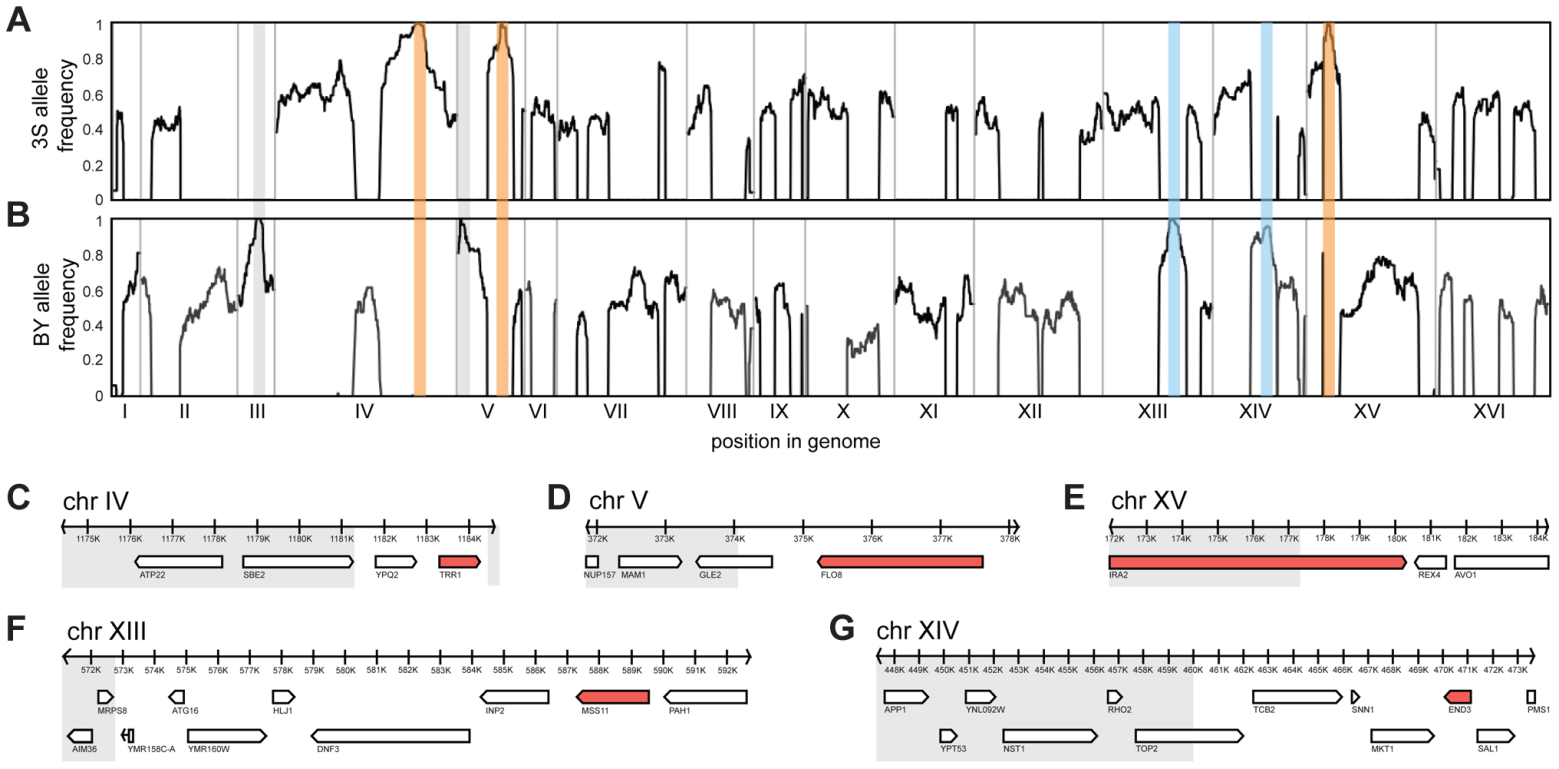
Genetic dissection of the five-way interaction. Genome-wide allele frequency plots are shown for mapping populations from backcrosses to BY (A) and 3S (B). Enriched loci are outlined in orange (causal allele from 3S parent), blue (causal allele from BY parent), or grey (allele is a selectable marker engineered into BY). Genes within the Chromosome IV (C), V (D), XV (E), XIII (F), and XIV (G) loci are shown. Grey boxes delimit regions of the loci that were subsequently excluded from consideration based on fine-mapping experiments. All non-essential genes within these windows were deleted in a multi-locus introgression strain that showed rough morphology. Genes that caused loss of the phenotype when deleted are colored red.

To identify loci that contribute to rough morphology, we generated thousands of random spores from the aforementioned backcrosses and used low-coverage whole genome sequencing to selectively genotype individuals that showed the phenotype (Methods). We obtained 92 and 88 rough segregants from the BY and 3S backcrosses, respectively. Using these data, we detected five genomic loci that were strongly enriched among these individuals but not among control segregants (Figure S2): three on Chromosomes IV, V, and XV inherited from 3S (Figure 2A), and two on Chromosomes XIII and XIV inherited from BY (Figure 2B). All of these loci, except the one on Chromosome XIV, were fixed among individuals with rough morphology.

We attempted to determine causal genes underlying each of the five loci. Our initial resolution of the loci was between 4 and 14 genes (Figure 2C–G; Table S3; Methods). To decrease the number of candidate genes, we performed targeted genotyping on 19 additional backcross segregants, as well as 8 multi-locus introgression strains that had been subjected to 6 rounds of backcrossing with selection for the rough phenotype (Figure S3; Methods). This additional stage of genetic mapping refined the loci to between 2 and 9 genes per locus, and 22 genes in total (Figure 2C–G; Table S4, S5, S6). We deleted each of the 20 remaining non-essential candidate genes from one of the multi-locus introgression strains (Methods). Across these deletions, a single gene at each locus showed an effect on the phenotype: *TRR1* (Chromosome IV), *FLO8* (Chromosome V), *MSS11* (Chromosome XIII), *END3* (Chromosome XIV), and *IRA2* (Chromosome XV) (Figure 2C–G). Because the two remaining candidate genes—*AVO1* and *TOP2*—were essential, we examined them using an alternative strategy that suggested they do not contribute to the observed colony morphology variation (Text S1).

**Figure 3:**
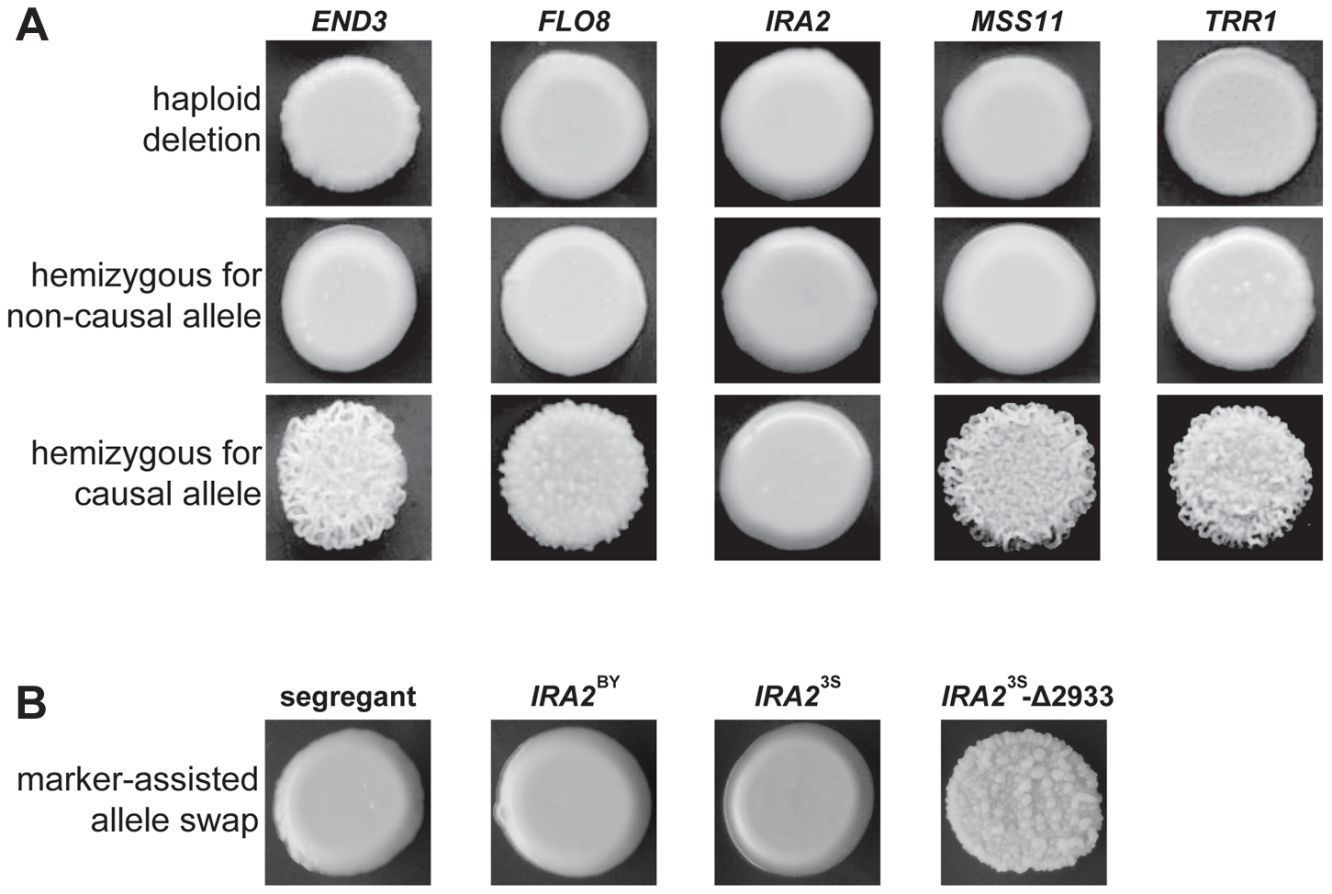
Evidence for functional variation at identified genes. In A, we show representative results from genetic engineering experiments in a multi-locus introgression strain. Haploid deletion strains are shown in the top row, while the second and third rows contain diploid hemizygotes. For each gene and allele, we constructed multiple hemizygotes, with representative phenotypes shown in the figure. The terms ‘causal’ and ‘non-causal’ refer to which allele was detected in our initial genetic mapping experiment. In B, we used genetic engineering in a smooth backcross segregant with the genotype *END3* ^BY^ *FLO8* ^3S^ *IRA2* ^BY^ *MSS11* ^BY^ *TRR1* ^3S^ to confirm the involvement of *IRA2* in rough colony morphology. The segregant subjected to transformations is shown on the left, followed by representative knock-ins of *IRA2* ^BY^, *IRA2* ^3S^, and *IRA2* ^3S^-Δ2933. The allele replacements have a *kanMX* tail, which was used to select for integration into the chromosome.

We used complementation tests to determine whether the five identified genes possess functional variation (Methods). Each haploid deletion strain was mated to three rough and three smooth haploid backcross progeny (Methods). These matings were designed to produce diploids that were homozygous for the required alleles at four of the causal loci and hemizygous for the fifth causal locus. For *END3*, *FLO8*, *MSS11*, and *TRR1*, the experiments provided support that the parental alleles differ in their effects. All matings of deletion strains to smooth backcross progeny produced smooth hemizygotes. Further, either two (in the cases of *FLO8* and *MSS11*) or three (in the cases of *TRR1* and *END3*) of the matings of deletion strains to rough backcross progeny produced rough hemizygotes (Figure 3A). However, for *IRA2*, the two possible hemizygotes showed no phenotypic difference, with both exhibiting smooth morphology (Figure 3A). *IRA2* has been reported to show haploinsufficiency in growth rate experiments [20], and this haploinsufficiency may also explain some of our reciprocal hemizygosity results for this gene.

To provide stronger support for *IRA2*'s role in the trait, we performed allele replacements of *IRA2* in a smooth backcross segregant that carried the non-causal allele of *IRA2*, as well as the causal alleles of *END3*, *FLO8*, *MSS11*, and *TRR1* (Methods). While transformations with the *IRA2* ^3S^ allele had no phenotypic effect, we found that transformations with the *IRA2* allele from the rough segregant that had been backcrossed resulted in a change from smooth to rough morphology (Figure 3B). Sequencing of *IRA2* from 3S and the rough segregant revealed a single difference between the two alleles: a frameshift mutation that truncates the protein by 117 amino acids (hereafter referred to as *IRA2* ^3S^-Δ2933; Text S2). *IRA2* is known to be hypermutable and spontaneous mutations in this gene have been shown to influence a variety of multicellular growth phenotypes [19], [21]. However, our results demonstrate that the effects of spontaneous mutations in *IRA2* can depend on an individual's genotype at a number of additional genes. We also checked for *IRA2* ^3S^-Δ2933 in the four other rough individuals that we found in our original BY×3S mapping population. Three of these rough segregants possessed the frameshift mutation, suggesting that *IRA2* ^3S^-Δ2933 probably arose during the outgrowth of the BY/3S diploid prior to its sporulation.

Previous work by other groups identified functional polymorphisms in *END3* and *FLO8* that also segregate in our cross [22], [23]. BY has a premature stop mutation in *FLO8* that prevents it from undergoing many forms of multicellular growth [22]. As for *END3*, a missense polymorphism in this gene contributes to variability in high temperature growth in a cross of the clinical isolate YJM789 and S288c, the progenitor of BY [23]. Of relevance to our study, this variant in *END3* has effects that are strongly dependent on genetic background [24]. With respect to *TRR1*, the *Saccharomyces* Genome Resequencing Project [25] and our own sequencing data indicate that the BY and 3S alleles of this gene differ by a single nucleotide, which is a synonymous SNP in the 52^nd^ codon of the gene: BY has an ATC codon and 3S has an ATT codon. Although both of these codons are recognized by the same isoleucine tRNA, the ATT codon is preferred by a nearly two-to-one ratio throughout the yeast genome, suggesting that the SNP might have an effect on translational efficiency. Only lab-derived *S. cerevisiae* strains carry the ATC allele that confers smooth morphology, while all other sequenced *S. cerevisiae* and *S. paradoxus* strains harbor the ATT allele that is likely involved in rough morphology. Work to determine the functional variant(s) in *MSS11*, which possesses a number of coding and noncoding polymorphisms that could have effects, is ongoing (Table S7).

The causal genes encode proteins with diverse cellular functions: End3 plays a role in clathrin-mediated endocytosis [26], [27], Flo8 and Mss11 are transcription factors that regulate cell-cell adhesion and multicellular phenotypes in *S. cerevisiae* [28], [29], Ira2 is a negative regulator of the RAS-cAMP pathway [30], and Trr1 is an enzyme involved in oxidative stress response [31], [32]. Flo8 and Mss11 physically interact [33], and *IRA2* and *MSS11* show a genetic interaction when both are knocked out [34]. To our knowledge, none of the other pairs of identified genes have been reported to interact at the biochemical, genetic, physical, or regulatory levels. To assess whether Flo8 and Mss11 might directly regulate the expression of the other genes, we examined existing data from calling card analyses, a technique that identifies genomic sites bound by transcription factors [16]. These results indicated that Flo8 and Mss11 are unlikely to bind the promoters of *END3*, *IRA2*, and *TRR1*, although admittedly the study involved a different strain than our cross parents.

**Figure 4:**
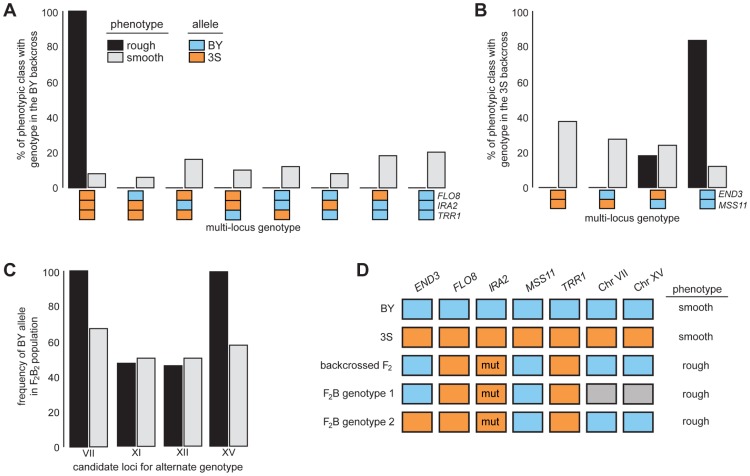
Segregation analysis of causal genes and identification of loci that complement *END3* ^3S^. Spores from dissected tetrads were phenotyped and then genotyped at *END3*, *FLO8*, *IRA2*, *MSS11*, and *TRR1*. Results from the BY and 3S backcrosses are in A and B, respectively. In C, we show genotyping results for candidate loci on Chromosomes VII, XI, XII, and XV in a population of *END3* ^3S^ segregants from a second-generation backcross. Lastly, in D, we show the different genotypes in the BY×3S cross that specify rough morphology. In A–D, blue and orange denote BY and 3S alleles, respectively, while grey in D indicates that either allele can occur. All rough individuals incorporated into this figure possessed *IRA2* ^3S^-Δ2933. The mutant allele is denoted in D by the label ‘mut’. Rough and smooth morphology are specified in A–C by black and light grey, respectively.

After identifying causal genes at the five loci, we analyzed the effects of these genes in more detail by genotyping them in a panel of phenotyped segregants from dissected backcross tetrads (Methods). Every individual with rough morphology possessed the 3S allele of *FLO8* and *TRR1*, the BY allele of *MSS11*, and *IRA2* ^3S^-Δ2933 (Figures 4A–B and S4A–B; Tables S8 and S9). Although most individuals with rough morphology carried *END3* ^BY^, a small fraction of individuals with *END3* ^3S^ also showed the trait (Figures 4B and S4C; Table S9), indicating that alleles at additional loci complement *END3* ^3S^.

We more deeply investigated the genetic basis of rough morphology among individuals with *END3* ^3S^. First, we used a gene knockout strategy to check whether *END3* ^3S^ is necessary for these individuals to exhibit rough morphology (Methods). *end3* ^3S^ *Δ* strains were smooth (Figure S5), suggesting that the alternate genetic architecture for rough morphology requires *END3* ^3S^. Second, we tried to identify loci that complement *END3* ^3S^. Four rough *END3* ^3S^ progeny were present in our sequenced mapping population from the 3S backcross. Among these segregants, we detected 11 previously unidentified genomic regions where individuals shared the same genotype (Figure S6; Table S10; Methods). We were able to reduce this set to four candidate loci on Chromosomes VII, XI, XII, and XV by genotyping additional backcross progeny (Table S11; Methods). To determine which of the four loci have causal roles in rough morphology, we mated a relevant backcross segregant to 3S and analyzed a panel of 51 second-generation backcross progeny (Table S12; Methods). The BY alleles at the Chromosome VII and XV loci were fixed among the 39 individuals with rough morphology, while the other two loci showed no evidence of playing a role in the trait (Figure 4C; Table S12). Given only individuals that carried BY alleles at both the Chromosome VII and XV loci exhibited rough morphology, it is likely that these loci genetically interact to complement *END3* ^3S^.

Our findings indicate that the segregant used for backcrossing carried more than one set of interacting alleles that can specify rough morphology (Figure 4D). Identifying the causal genes and genetic variants underlying the Chromosome VII and XV loci can thus shed light on how these different genotypes produce the same trait. However, our ongoing efforts to clone the causal factors at these loci are limited by the crude resolution of the present data (each locus is presently resolved to >60 kilobases; Table S10). We note that initial gene deletion experiments focused on 18 candidates (Table S13), including *LAS17* and *YAP1802*, whose cognate proteins functionally interact with End3 [35], [36], have been unsuccessful. Moving forward, we plan to determine the genes that underlie the Chromosome VII and XV loci, and characterize their relationship with *END3*.

In summary, we have demonstrated that sets of five or more genetic variants can synthetically interact to produce major phenotypic effects. Alleles involved in these higher-order interactions may either be polymorphisms that segregate in natural populations or spontaneous mutations. Our results also illustrate that rather than functioning in a single biochemical pathway, protein complex, or regulatory circuit, the genes involved in higher-order interactions can play roles in a number of cellular processes. This finding implies that characterizing higher-order interactions using data from screens and annotations focused solely on reference genomes may be a challenge, and highlights how genetic variation can serve as a tool for detecting previously unidentified functional relationships among genes. Further, we have shown that multiple sets of alleles can interact to produce the same phenotypic effect. Additional work is necessary to determine how this latter finding is mediated at the molecular and systems levels. Overall, our study suggests that characterizing the larger-scale contribution of higher-order interactions to phenotypic variation is a necessary step in improving our basic understanding of the genotype-phenotype map.

## Methods

### Phenotyping of yeast colony morphology

All phenotyping experiments were performed on agar plates containing yeast extract and peptone (YP) with 2% ethanol as the carbon source (YPE). Prior to phenotyping, strains were grown up in liquid YP with 2% dextrose (YPD). Stationary-phase cultures were manually pinned onto YPE and allowed to grow for five days at 30°C, and were then imaged using a standard digital camera.

### Assessing potential effects of transient heritable factors

Sequencing data from the rough segregant used in backcross experiments was examined at the chromosome-scale for evidence of aneuploidy. Average per base coverage of each chromosome was computed in R and compared to the genome-wide average. This segregant was also plated at low density on a large number of YPE plates. We screened tens of thousands of colonies for instances of phenotypic switching and observed no cases where an individual converted from rough to smooth morphology.

### Generation of backcross segregants

Strains used in this paper contained the Synthetic Genetic Array marker system [37], which allowed us to easily generate large numbers of recombinant *MAT* ***a*** progeny. All segregants discussed in the paper were *MAT* ***a*** *can1Δ::STE2pr-SpHIS5 his3Δ* and all backcrosses involved mating these individuals to either a BY or a 3S strain that was *MATα his3Δ*. In these crosses, strains with opposite mating types were mixed together on a YPD plate and incubated for four hours at 30°C. Zygotes were then obtained by microdissection. To generate segregants, diploids were sporulated at room temperature using the protocol described by Guthrie and Fink [38]. Once sporulation had completed, spore cultures were digested with β-glucuronidase and then plated onto yeast nitrogen base (YNB) plates containing canavanine, as described previously [39]. Spores were plated at a density of roughly 100 to 200 colonies per plate.

### Genome sequencing of backcross segregants

Whole genome sequencing libraries were prepared using the Illumina Nextera kit, with each of the backcross segregants barcoded with a unique sequence tag. The libraries were mixed together in equimolar fractions and sequenced on an Illumina HiSeq machine by the Beijing Genomics Institute using 100 base pair (bp) ×100 bp reads. Sequencing reads were then mapped to the *S. cerevisiae* reference genome using the Burrows-Wheeler Aligner (BWA) [40]. We used data from 36,756 high confidence SNPs that had been identified based on comparison of Illumina sequence data for 3S to the BY genome. Similar to Andolfatto et al. [41], we employed Hidden Markov Models (HMMs) to determine the haplotypes of the segregants based on the sequencing data. We computed the fraction of reads at each SNP that came from BY and used the vector of these fractions in HMMs that were implemented chromosome-by-chromosome in the HMM() package of the R statistical programming environment. Any segregants producing data that showed evidence of contamination, diploidy, or aneuploidy were excluded from genetic mapping and downstream analyses. Four and eight such individuals were left out of the BY and 3S mapping populations, respectively.

### Genetic mapping

Genotypes inferred from the HMM were used in genetic mapping analyses. At each position in the genome, we determined the fraction of individuals that carried the allele from the parent not used in the backcross. We scanned the genome for alleles from the non-backcross parent that were detected in a large fraction of segregants. We report loci where these alleles were at 95% frequency or higher. To determine intervals in which causal genes were located, we identified the smallest region that was bounded by recombination breakpoints among individuals from a backcross that shared the same allele at a peak.

### Generation and genotyping of dissected tetrads

Backcross diploids were sporulated and digested in β-glucuronidase to permit tetrad dissection. Standard microdissection techniques were used to isolate tetrads and separate individual spores.

### Fine-mapping using multi-locus introgression strains and dissected backcross segregants

Haploid multi-locus introgression strains were constructed using six rounds of recurrent backcrossing with phenotypic selection, starting from the same segregant used in our backcross mapping experiment. Eight of these strains were generated, with four made by recurrently backcrossing to 3S and four made by recurrently backcrossing to BY. We also used a subset of individuals from the tetrad dissections that showed rough morphology. To conduct the fine-mapping, we typed these individuals at a number of markers in each interval using PCR and restriction digestion, or Sanger sequencing.

### Genetic engineering experiments

All genes within causal loci were deleted using the CORE cassette, in the same manner described by Storici et al. [42]. Homology tails matching the 60 bases immediately up- and downstream of each gene were attached to the CORE cassette through PCR and introduced into cells using the Lithium Acetate method [43]. Selection for G418 resistance was used to screen for integration of the CORE cassette; correct integration was then checked using PCR. All deletions were performed in a haploid multi-locus introgression strain. To perform complementation tests, deletion strains were mated to multiple dissected segregants that carried either the causal or non-causal allele of the deleted gene, as well as the causal alleles at the four other involved genes. The same phenotyping methods described above were employed for these strains. To generate allele replacement strains for *IRA2*, a smooth segregant with the non-causal allele of *IRA2* and the causal alleles at the other four loci was transformed using a modified form of adaptamer mediated allele replacement [44]. Transformations were conducted with two partially overlapping PCR products—a full-length amplicon of *IRA2* that was tailed at the 3′ end with the 5′ portion of the *kanMX* cassette and a copy of the *kanMX* cassette that was tailed on the 3′ end with part of the intergenic region downstream of *IRA2*. Knock-ins were identified using selection on G418 and verified by Sanger sequencing.

### Identification of loci that complement the 3S allele of *END3*

Sequenced strains from the backcross to 3S were partitioned based on their genotype at *END3*. We then screened these individuals for sites where they all carried BY alleles. A group of additional rough segregants with *END3* ^3S^ that had been obtained during tetrad dissections were genotyped by PCR amplification and restriction digestion of markers across each of the new loci. One of these additional backcross segregants was mated to 3S, and a panel of rough progeny from this second-generation backcross were typed at the remaining candidate loci.

## Supporting Information

Figure S1 Sequencing coverage for each chromosome of the backcrossed rough segregant. The segregant used in backcrossing was sequenced to ∼4.39× coverage. We determined the average coverage of nucleotides on each chromosome (grey bars) and across the genome (dotted line). None of the chromosomes exhibited a significant excess or deficit of coverage, suggesting that the strain did not carry any large aneuploidies.
(PDF)

Figure S2 Allele frequency plots for control populations of BY and 3S backcross segregants. To account for unintentional selection in our mapping populations, we sequenced a control population of segregants from each backcross. Genome-wide allele frequency plots are shown for control populations of segregants from backcrosses to BY (A) and 3S (B). The diploid parent of these backcrosses was sporulated and plated at high density on selective medium to obtain recombinant *MAT* ***a*** backcross progeny (Methods). Thousands of segregants were pooled together by scraping them off plates. DNA was extracted from the pools and used to generate Illumina whole genome sequencing libraries. These libraries were then sequenced to ∼200× coverage. We pulled out data for SNPs described in the Methods and used these data to generate the above plots. The plot was generated by smoothing the data for each chromosome using the filter() function in R and a window size of 50 SNPs. Causal loci for rough morphology are labeled with black arrows and selected markers used to generate *MAT* ***a*** progeny are labeled with grey arrows. We note that there is a site on Chromosome XIV near the causal locus that shows enrichment, but is distinct from the region involved in rough morphology.
(PDF)

Figure S3 Generation of multi-locus introgression strains. A rough segregant was subjected to six rounds of backcrossing with selection for the rough phenotype to reduce the genetic contribution of one parent strain and allow for finer resolution of causal loci.
(PDF)

Figure S4 Segregating phenotypes observed in cross between BY and 3S. The phenotypes of representative recombinant genotypes are shown: (A) a rough segregant obtained from the backcross to 3S, (B) a rough segregant obtained from the backcross to BY, (C) an individual with the 3S allele at *END3* that shows rough morphology, (D) an individual with the BY allele at *TRR1* but the interacting alleles at *END3*, *FLO8*, *IRA2*, and *MSS11* shows a bumpy surface, (E) a smooth segregant from the backcross to 3S, and (F) a smooth segregant from the backcross to BY.
(PDF)

Figure S5 Smooth phenotype of *end3* ^3S^ *Δ* individual. *END3* was deleted from a segregant that possessed the *END3* ^3S^ allele and the alleles that cause rough morphology at *TRR1*, *FLO8*, *MSS11*, *AVO1*, and the additional loci on chromosomes VII and XV.
(PDF)

Figure S6 Allele frequencies among rough individuals with *END3^3S^*. Allele frequencies of individuals from the initial 3S mapping population that showed rough morphology but lacked the BY allele of *END3* are plotted. Additional 3S backcross segregants were obtained, and analyzed as individuals using phenotyping and genotyping at the new loci. The SGA markers used to select *MAT* ***a*** haploid segregants are labelled with a grey arrow, while the chromosome XIII locus containing *MSS11* is labelled with a green arrow. The 11 novel loci are shown with black and red arrows, with the difference being black loci were sites that remained as candidates after typing of additional segregants that possessed rough morphology and the alternate causal genotype.
(PDF)

Table S1 Phenotypes and genotypes of tetrad spores from the backcross to BY. Individuals from 14 dissected tetrads were phenotyped and genotyped at segregating markers within causal loci. Phenotypes are recorded as smooth (s), rough (r) or bumpy subphenotype (b). Genotypes at segregating markers within each locus are denoted as 1 (3S) or 0 (BY). Under the column “Spore #”, the number in the name represents the tetrad, while the letters signify different spores.
(DOCX)

Table S2 Phenotypes and genotypes of tetrad spores from the backcross to 3S. Individuals from 13 dissected tetrads were phenotyped and genotyped at segregating markers within causal loci. Phenotypes are recorded as smooth (s) or rough (r). Genotypes at segregating markers within each locus are denoted as 1 (BY) or 0 (3S). Under the column “Spore #”, the number in the name represents the tetrad, while the letters signify different spores. 5 individuals possessed BY alleles at *MSS11* and *END3*, yet showed smooth morphology. Further genotyping revealed that bolded individuals lacked the *IRA2* ^3S^-Δ2933 allele.
(DOCX)

Table S3 Initial bounds of detected loci for the five-way interaction. Causal loci were defined as regions present in at least 95% of individuals. To identify the intervals at these loci, we took all individuals with the causal allele and determined the minimum region delimited by recombination breakpoints.
(DOCX)

Table S4 Genotyping of multi-locus introgressed lines in the BY direction. Multi-locus introgressed lines were generated through six rounds of backcrossing to BY with phenotypic selection (Methods; Figure S2). Lines were genotyped across causal loci by Sanger Sequencing of segregating markers. 3S alleles are denoted as ‘1’; BY alleles are denoted as ‘0’.
(DOCX)

Table S5 Genotyping of multi-locus introgressed strains in the 3S direction. Multi-locus introgressed lines were generated through six rounds of backcrossing to 3S with phenotypic selection (Methods; Figure S2). Lines were genotyped across causal loci by Sanger Sequencing of segregating markers. BY alleles are denoted as ‘1’; 3S alleles are denoted as ‘0’.
(DOCX)

Table S6 Further genotyping of tetrad spores from the 3S backcross. Select tetrad spores from the 3S backcross were genotyped across causal loci by Sanger Sequencing of segregating markers. A 1 indicates that genotyped individuals possessed the 3S allele at a given marker and 0 indicates the BY allele.
(DOCX)

Table S7 SNPs detected within and near causal genes. Each SNP detected between BY and 3S detected in our sequencing is listed along with its protein sequence outcome if it is nonsynonymous.
(DOCX)

Table S8 Genotypes within each phenotypic class among tetrad spores from the backcross to BY.
(DOCX)

Table S9 Genotypes within each phenotypic class among tetrad spores from the backcross to 3S.
(DOCX)

Table S10 Bounds of fixed loci among rough individuals with *END3^3S^*. Four sequenced segregants with rough morphology possessed *END3^3S^*. These individuals shared 11 previously undetected loci. Intervals we later identify as causal are in bold.
(DOCX)

Table S11 Genotyping of additional rough individuals possessing *END3^3S^*. Two rough individuals with *END3^3S^* were typed across loci identified in Figure S6. A 1 indicates that all genotyped individuals possessed the BY allele at a given marker and 0 indicates the 3S allele.
(DOCX)

Table S12 Genotyping of rough *END3^3S^*×3S second-generation backcross segregants. 39 individuals with rough morphology (1–39) and 12 with smooth morphology (c1–c12) were genotyped at segregating markers within four candidate loci. A 1 indicates that all genotyped individuals possessed the 3S allele at a given marker and 0 indicates the BY allele.
(DOCX)

Table S13 Genes within additional loci tested by deletion. Each of the above genes were tested for involvement in rough morphology by deletion in a rough individual.
(DOCX)

Text S1 Evaluation of the essential genes *AVO1* and *TOP2*.
(DOCX)

Text S2 Protein sequences of Ira2^BY^, Ira2^3S^, and Ira2^3S^ -Δ2933.
(DOCX)
