# Supplementary figures and images for: Genetic Interactions Involving Five or More Genes Contribute to a Complex Trait in Yeast
Source: PLoS Genet. 2014 May 1;10(5):e1004324. doi: 10.1371/journal.pgen.1004324 (PMC4006734; doi:10.1371/journal.pgen.1004324)

mean coverage

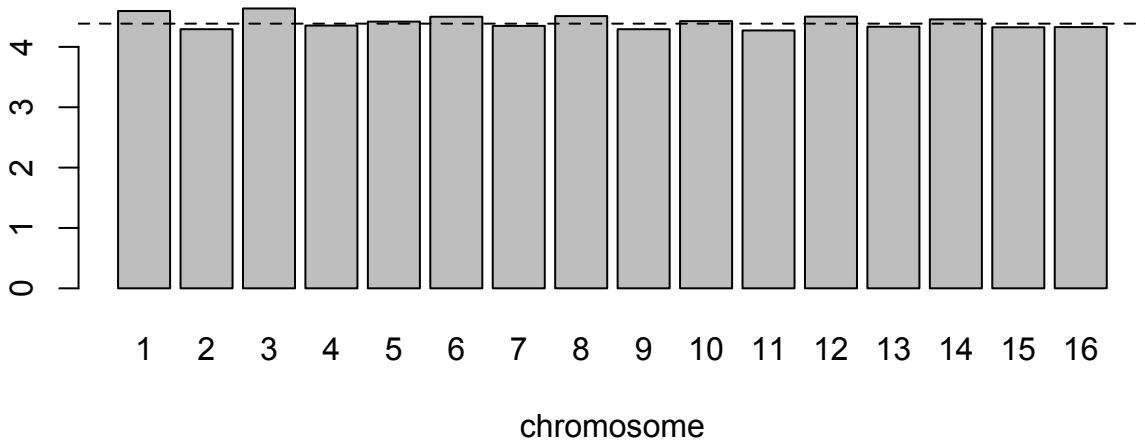

Supplement: Figure S1 — Sequencing coverage for each chromosome of the backcrossed rough segregant. The segregant used in backcrossing was sequenced to ∼4.39× coverage. We determined the average coverage of nucleotides on each chromosome (grey bars) and across the genome (dotted line). None of the chromosomes exhibited a significant excess or deficit of coverage, suggesting that the strain did not carry any large aneuploidies. (PDF) [file pgen.1004324.s001.pdf]

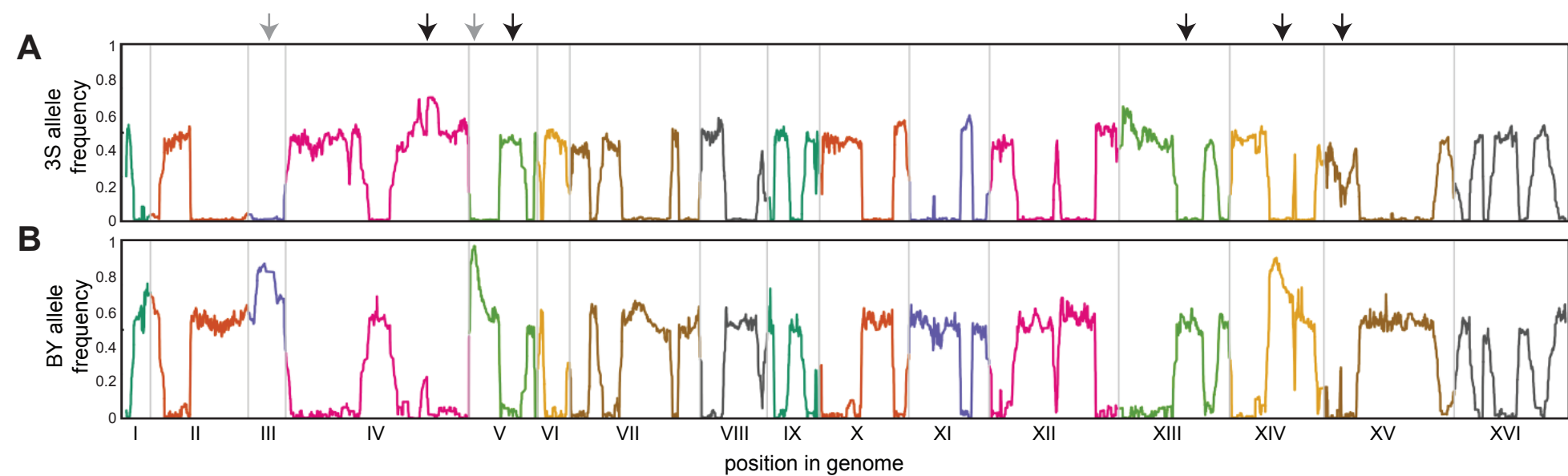

Supplement: Figure S2 — Allele frequency plots for control populations of BY and 3S backcross segregants. To account for unintentional selection in our mapping populations, we sequenced a control population of segregants from each backcross. Genome-wide allele frequency plots are shown for control populations of segregants from backcrosses to BY (A) and 3S (B). The diploid parent of these backcrosses was sporulated and plated at high density on selective medium to obtain recombinant MAT a backcross progeny (Methods). Thousands of segregants were pooled together by scraping them off plates. DNA was extracted from the pools and used to generate Illumina whole genome sequencing libraries. These libraries were then sequenced to ∼200× coverage. We pulled out data for SNPs described in the Methods and used these data to generate the above plots. The plot was generated by smoothing the data for each chromosome using the filter() function in R and a window size of 50 SNPs. Causal loci for rough morphology are labeled with black arrows and selected markers used to generate MAT a progeny are labeled with grey arrows. We note that there is a site on Chromosome XIV near the causal locus that shows enrichment, but is distinct from the region involved in rough morphology. (PDF) [file pgen.1004324.s002.pdf]

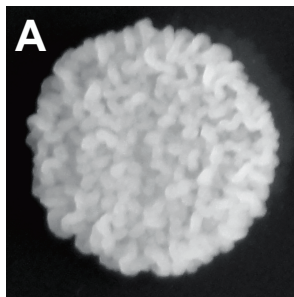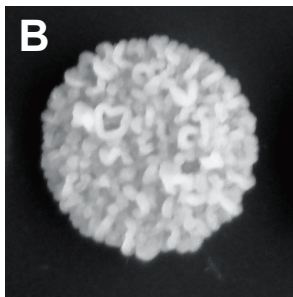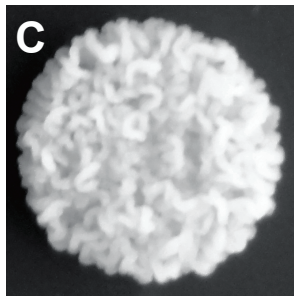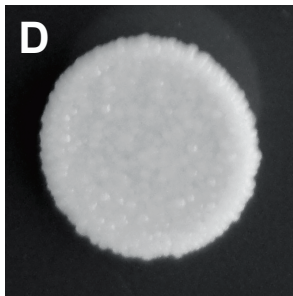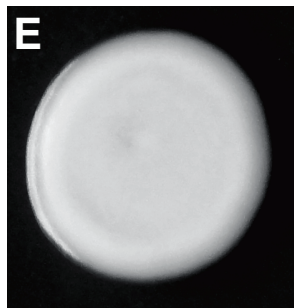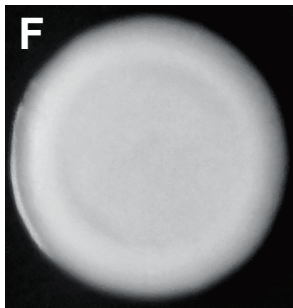

Supplement: Figure S4 — Segregating phenotypes observed in cross between BY and 3S. The phenotypes of representative recombinant genotypes are shown: (A) a rough segregant obtained from the backcross to 3S, (B) a rough segregant obtained from the backcross to BY, (C) an individual with the 3S allele at END3 that shows rough morphology, (D) an individual with the BY allele at TRR1 but the interacting alleles at END3, FLO8, IRA2, and MSS11 shows a bumpy surface, (E) a smooth segregant from the backcross to 3S, and (F) a smooth segregant from the backcross to BY. (PDF) [file pgen.1004324.s004.pdf]

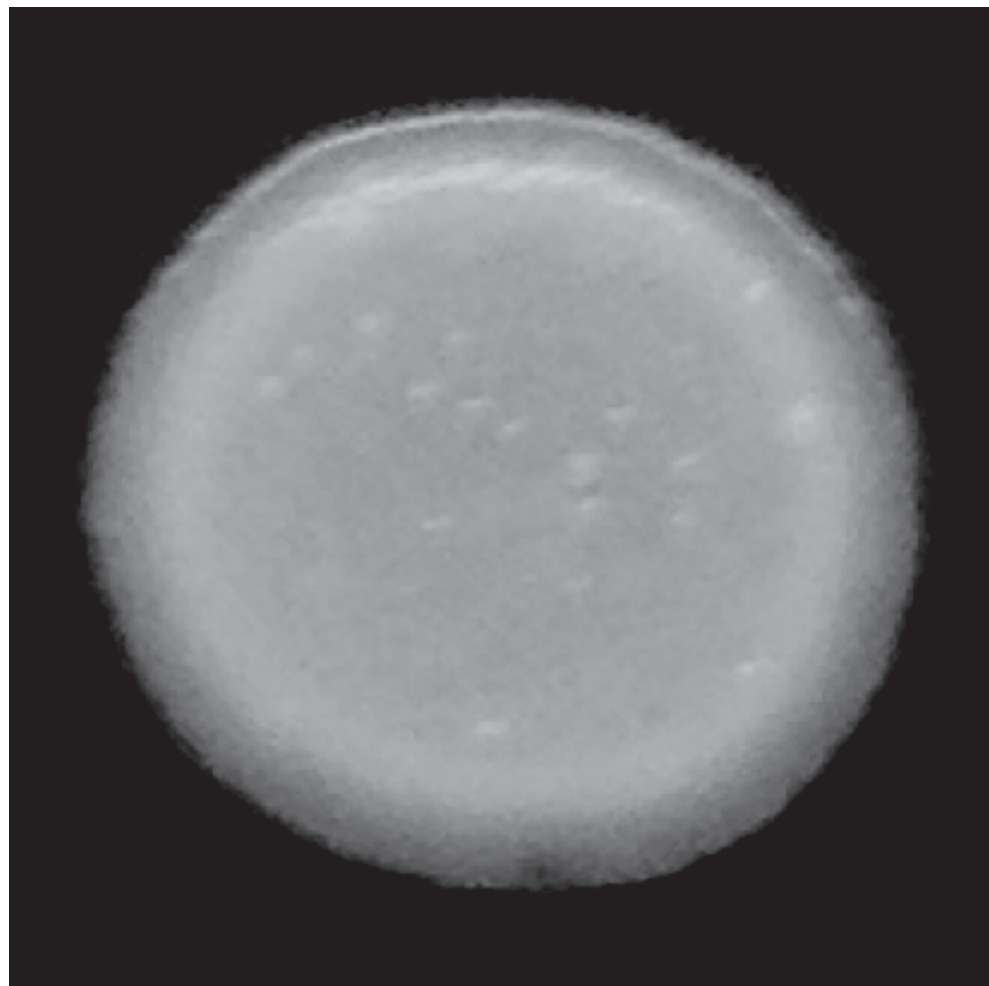

Supplement: Figure S5 — Smooth phenotype of end3 3S Δ individual. END3 was deleted from a segregant that possessed the END3 3S allele and the alleles that cause rough morphology at TRR1, FLO8, MSS11, AVO1, and the additional loci on chromosomes VII and XV. (PDF) [file pgen.1004324.s005.pdf]

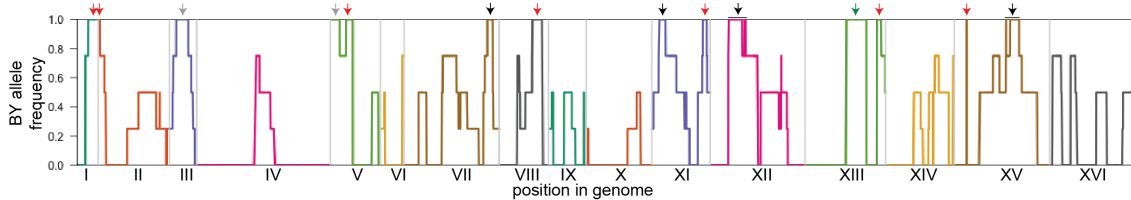

Supplement: Figure S6 — Allele frequencies among rough individuals with END33S. Allele frequencies of individuals from the initial 3S mapping population that showed rough morphology but lacked the BY allele of END3 are plotted. Additional 3S backcross segregants were obtained, and analyzed as individuals using phenotyping and genotyping at the new loci. The SGA markers used to select MAT a haploid segregants are labelled with a grey arrow, while the chromosome XIII locus containing MSS11 is labelled with a green arrow. The 11 novel loci are shown with black and red arrows, with the difference being black loci were sites that remained as candidates after typing of additional segregants that possessed rough morphology and the alternate causal genotype. (PDF) [file pgen.1004324.s006.pdf]
