# Supplementary material for: Genetic Interactions Involving Five or More Genes Contribute to a Complex Trait in Yeast
Source: PLoS Genet. 2014 May 1;10(5):e1004324. doi: 10.1371/journal.pgen.1004324 (PMC4006734; doi:10.1371/journal.pgen.1004324)
Supplement: Figure S3 — Generation of multi-locus introgression strains. A rough segregant was subjected to six rounds of backcrossing with selection for the rough phenotype to reduce the genetic contribution of one parent strain and allow for finer resolution of causal loci. (PDF) [file pgen.1004324.s003.pdf]

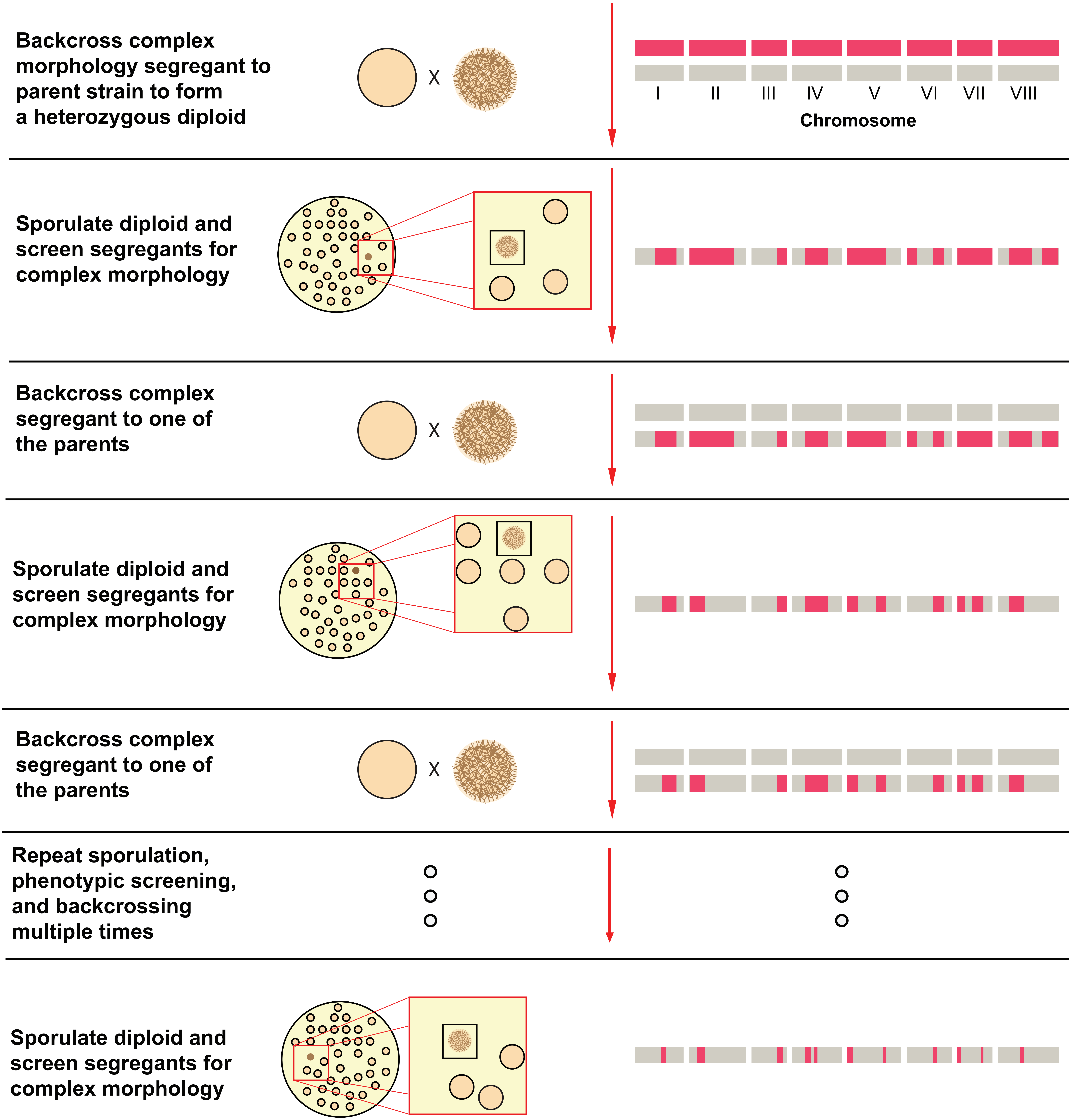
Backcross complex morphology segregant to parent strain to form a heterozygous diploid
X
I
II
III
IV
V
VI
VII
VIII
Chromosome
Sporulate diploid and screen segregants for complex morphology
Backcross complex segregant to one of the parents
X
Sporulate diploid and screen segregants for complex morphology
Backcross complex segregant to one of the parents
X
Repeat sporulation, phenotypic screening, and backcrossing multiple times
Sporulate diploid and screen segregants for complex morphology
