# Supplementary material for: Genetic Interactions Involving Five or More Genes Contribute to a Complex Trait in Yeast
Source: PLoS Genet. 2014 May 1;10(5):e1004324. doi: 10.1371/journal.pgen.1004324 (PMC4006734; doi:10.1371/journal.pgen.1004324)
Supplement: Table S1 — Phenotypes and genotypes of tetrad spores from the backcross to BY. Individuals from 14 dissected tetrads were phenotyped and genotyped at segregating markers within causal loci. Phenotypes are recorded as smooth (s), rough (r) or bumpy subphenotype (b). Genotypes at segregating markers within each locus are denoted as 1 (3S) or 0 (BY). Under the column “Spore #”, the number in the name represents the tetrad, while the letters signify different spores. (DOCX) [file pgen.1004324.s007.docx]

| Spore # | chrIV | chrV | chrXV | mat | phenotype |
| --- | --- | --- | --- | --- | --- |
| 1A | 1 | 1 | 0 | x | s |
| 1B | 1 | 1 | 1 | a | b |
| 1C | 0 | 0 | 0 | a | s |
| 1D | 0 | 0 | 1 | x | s |
| 2A | 0 | 0 | 1 | a | s |
| 2B | 0 | 0 | 0 | a | s |
| 2C | 1 | 1 | 1 | x | r |
| 2D | 1 | 1 | 0 | x | s |
| 3A | 0 | 0 | 1 | a | s |
| 3B | 1 | 1 | 0 | x | s |
| 3C | 0 | 0 | 0 | x | s |
| 3D | 1 | 1 | 1 | a | r |
| 4A | 0 | 1 | 1 | a | b |
| 4B | 1 | 1 | 1 | x | r |
| 4C | 1 | 0 | 0 | a | s |
| 4D | 0 | 0 | 0 | x | s |
| 5A | 0 | 0 | 1 | x | s |
| 5B | 1 | 0 | 0 | x | s |
| 5C | 0 | 1 | 0 | a | s |
| 5D | 1 | 1 | 1 | a | s |
| 6A | 1 | 1 | 0 | a | s |
| 6B | 0 | 1 | 1 | x | b |
| 6C | 0 | 0 | 0 | x | s |
| 6D | 1 | 0 | 1 | a | s |
| 7A | 0 | 0 | 0 | a | s |
| 7B | 1 | 0 | 1 | x | s |
| 7C | 1 | 1 | 0 | x | s |
| 7D | 0 | 1 | 1 | a | b |
| 8A | 1 | 1 | 1 | x | r |
| 8B | 0 | 0 | 1 | x | s |
| 8C | 1 | 1 | 0 | a | s |
| 8D | 0 | 0 | 0 | a | s |
| 9A | 1 | 0 | 1 | a | s |
| 9B | 0 | 0 | 1 | x | s |
| 9C | 1 | 1 | 0 | a | s |
| 9D | 0 | 1 | 0 | x | s |
| 10A | 0 | 0 | 1 | x | s |
| 10B | 1 | 0 | 0 | a | s |
| 10C | 1 | 1 | 1 | a | b |
| 10D | 0 | 1 | 0 | x | s |
| 11A | 1 | 0 | 0 | x | s |
| 11B | 0 | 1 | 1 | a | s |
| 11C | 1 | 1 | 1 | a | s |
| 11D | 0 | 0 | 0 | x | s |
| 12A | 0 | 0 | 1 | a | s |
| 12B | 1 | 1 | 1 | x | r |
| 12C | 1 | 1 | 0 | a | s |
| 12D | 0 | 0 | 0 | x | s |
| 13A | 1 | 1 | 1 | x | r |
| 13B | 0 | 0 | 1 | x | s |
| 13C | 1 | 0 | 0 | a | s |
| 13D | 0 | 1 | 0 | a | s |
| 14A | 0 | 0 | 0 | x | s |
| 14B | 0 | 1 | 1 | a | b |
| 14C | 1 | 0 | 0 | x | s |
| 14D | 1 | 1 | 1 | a | r |
