# Supplementary material for: Genetic Interactions Involving Five or More Genes Contribute to a Complex Trait in Yeast
Source: PLoS Genet. 2014 May 1;10(5):e1004324. doi: 10.1371/journal.pgen.1004324 (PMC4006734; doi:10.1371/journal.pgen.1004324)
Supplement: Table S2 — Phenotypes and genotypes of tetrad spores from the backcross to 3S. Individuals from 13 dissected tetrads were phenotyped and genotyped at segregating markers within causal loci. Phenotypes are recorded as smooth (s) or rough (r). Genotypes at segregating markers within each locus are denoted as 1 (BY) or 0 (3S). Under the column “Spore #”, the number in the name represents the tetrad, while the letters signify different spores. 5 individuals possessed BY alleles at MSS11 and END3, yet showed smooth morphology. Further genotyping revealed that bolded individuals lacked the IRA2 3S-Δ2933 allele. (DOCX) [file pgen.1004324.s008.docx]

| spore | chrXIII | chrXIV | mat | phenotype |
| --- | --- | --- | --- | --- |
| 1A | 0 | 1 | x | s |
| 1B | 1 | 0 | a | s |
| 1C | 0 | 0 | a | s |
| **1D** | **1** | **1** | **x** | **s** |
| 2A | 0 | 1 | x | s |
| 2B | 1 | 0 | a | s |
| 2C | 0 | 1 | x | s |
| 2D | 1 | 0 | a | r |
| 3A | 0 | 1 | x | s |
| 3B | 0 | 0 | a | s |
| 3C | 1 | 1 | a | r |
| 3D | 1 | 0 | x | s |
| 4A | 0 | 0 | a | s |
| 4B | 0 | 0 | x | s |
| 4C | 1 | 1 | x | s |
| 4D | 1 | 1 | a | r |
| 5A | 1 | 1 | x | r |
| 5B | 0 | 1 | a | s |
| 5C | 0 | 0 | a | s |
| 5D | 1 | 0 | x | s |
| 6A | 0 | 0 | a | s |
| 6B | 0 | 0 | a | s |
| 6C | 1 | 1 | x | r |
| 6D | 1 | 1 | x | r |
| 7A | 0 | 1 | a | s |
| 7B | 0 | 1 | x | s |
| 7C | 1 | 0 | x | s |
| 7D | 1 | 0 | a | s |
| 8A | 1 | 0 | a | s |
| 8B | 0 | 0 | x | s |
| 8C | 1 | 1 | a | s |
| 8D | 0 | 0 | x | s |
| 9A | 0 | 1 | a | s |
| 9B | 1 | 1 | x | r |
| 9C | 1 | 0 | a | s |
| 9D | 0 | 0 | x | s |
| 10A | 1 | 0 | a | s |
| 10B | 0 | 1 | x | s |
| 10C | 0 | 1 | x | s |
| 10D | 1 | 0 | a | s |
| 11A | 1 | 0 | a | r |
| 11B | 0 | 0 | x | s |
| **11C** | **1** | **1** | **a** | **s** |
| 11D | 0 | 1 | x | s |
| 12A | 0 | 0 | a | s |
| 12B | 1 | 1 | x | r |
| **12C** | **1** | **1** | **a** | **s** |
| 12D | 0 | 0 | x | s |
| 13A | 0 | 0 | a | s |
| 13B | 0 | 0 | x | s |
| 13C | 1 | 1 | x | r |
| 13D | 1 | 1 | a | r |
