# Supplementary material for: Genetic Interactions Involving Five or More Genes Contribute to a Complex Trait in Yeast
Source: PLoS Genet. 2014 May 1;10(5):e1004324. doi: 10.1371/journal.pgen.1004324 (PMC4006734; doi:10.1371/journal.pgen.1004324)
Supplement: Table S3 — Initial bounds of detected loci for the five-way interaction. Causal loci were defined as regions present in at least 95% of individuals. To identify the intervals at these loci, we took all individuals with the causal allele and determined the minimum region delimited by recombination breakpoints. (DOCX) [file pgen.1004324.s009.docx]

| chromosome | start position | stop position |
| --- | --- | --- |
| IV | 1174395 | 1183454 |
| V | 371849 | 378140 |
| XIII | 571144 | 592735 |
| XIV | 447324 | 473648 |
| XV | 171973 | 184291 |
