# Supplementary material for: Genetic Interactions Involving Five or More Genes Contribute to a Complex Trait in Yeast
Source: PLoS Genet. 2014 May 1;10(5):e1004324. doi: 10.1371/journal.pgen.1004324 (PMC4006734; doi:10.1371/journal.pgen.1004324)
Supplement: Table S4 — Genotyping of multi-locus introgressed lines in the BY direction. Multi-locus introgressed lines were generated through six rounds of backcrossing to BY with phenotypic selection (Methods; Figure S2). Lines were genotyped across causal loci by Sanger Sequencing of segregating markers. 3S alleles are denoted as ‘1’; BY alleles are denoted as ‘0’. (DOCX) [file pgen.1004324.s010.docx]

|  | **typed pools** | | | |
| --- | --- | --- | --- | --- |
| **examined marker** | **1** | **2** | **3** | **4** |
| 4_1 | 0 | 0 | 1 | 0 |
| 4_1.5 | 0 | 0 | 1 | 0 |
| 4_2 | 1 | 1 | 1 | 1 |
| 4_3 | 1 | 1 |  |  |
| 4_3.5 | 0 | 0 | 1 | 0 |
| 4_4 | 0 | 0 | 1 | 0 |
| 5_0a | 1 | 1 | 1 | 0 |
| 5_0b | 1 | 1 | 1 | 0 |
| 5_1a | 1 | 1 | 1 | 1 |
| 5_1 | 1 |  |  |  |
| 5_2 | 1 | 1 | 1 | 1 |
| 5_3 | 1 | 1 | 1 | 1 |
| 5_4a | 1 | 1 | 1 | 1 |
| 5_4_5 | 1 | 0 | 1 | 0 |
| 15_1 | 1 | 0 | 1 | 1 |
| 15_2 | 1 | 1 | 1 | 1 |
| 15_3 | 1 | 1 | 1 | 1 |
| 15_3.5 | 0 |  | 1 | 1 |
| 15_4 | 0 | 1 | 1 | 0 |
