# Supplementary material for: Genetic Interactions Involving Five or More Genes Contribute to a Complex Trait in Yeast
Source: PLoS Genet. 2014 May 1;10(5):e1004324. doi: 10.1371/journal.pgen.1004324 (PMC4006734; doi:10.1371/journal.pgen.1004324)
Supplement: Table S6 — Further genotyping of tetrad spores from the 3S backcross. Select tetrad spores from the 3S backcross were genotyped across causal loci by Sanger Sequencing of segregating markers. A 1 indicates that genotyped individuals possessed the 3S allele at a given marker and 0 indicates the BY allele. (DOCX) [file pgen.1004324.s012.docx]

| Spore # | phenotype | 14_0 | 14_1 | 14_2 | 14_3 | 14_4 | 14_7 | 14_9 | 14_10 |
| --- | --- | --- | --- | --- | --- | --- | --- | --- | --- |
| 1D | S | 1 | 1 |  | 1 |  | 1 | 1 | 1 |
| 3C | R | 1 | 1 |  | 1 |  | 1 | 1 | 1 |
| 4D | R | 1 | 1 |  | 1 |  | 1 | 1 | 1 |
| 4C | S | 1 | 1 |  | 1 |  | 1 | 1 | 1 |
| 5A | R | 1 | 1 | 1 | 1 | 1 | 1 | 1 | 1 |
| 6C | R | 1 | 1 |  | 1 |  | 1 | 1 | 1 |
| 6D | R | 1 | 1 |  | 1 |  | 1 | 1 | 1 |
| 7D | S | 1 | 1 | 1 | 0 |  | 0 | 0 | 1 |
| 9B | R | 1 | 1 |  | 1 |  | 1 | 1 | 1 |
| 11C | S | 1 | 1 |  | 1 |  | 1 | 1 | 1 |
| 12B | R | 1 | 1 |  | 1 |  | 1 | 1 | 1 |
| 12C | S | 1 | 1 |  | 1 |  | 1 | 1 | 1 |
| 13C | R | 1 | 1 |  | 1 |  | 1 | 1 | 1 |
| 2D | R | 0 | 0 | 0 | 0 | 0 | 0 | 0 | 0 |
| 11A | R | 0 | 0 | 0 | 0 | 0 | 0 | 0 | 0 |
