# Supplementary material for: Genetic Interactions Involving Five or More Genes Contribute to a Complex Trait in Yeast
Source: PLoS Genet. 2014 May 1;10(5):e1004324. doi: 10.1371/journal.pgen.1004324 (PMC4006734; doi:10.1371/journal.pgen.1004324)
Supplement: Table S7 — SNPs detected within and near causal genes. Each SNP detected between BY and 3S detected in our sequencing is listed along with its protein sequence outcome if it is nonsynonymous. (DOCX) [file pgen.1004324.s013.docx]

| Gene | Chromosome | SNP position | BY allele | 3S allele | BY amino acid | 3S amino acid | Codon position |
| --- | --- | --- | --- | --- | --- | --- | --- |
| *TRR1* | 4 | 1183454 | C | T |  |  |  |
| *FLO8* | 5 | 376468 | A | C | Cys | Gly | 382 |
| *FLO8* | 5 | 377190 | T | C | * | Trp | 141 |
| *MSS11* | 13 | 587493 | T | G |  |  |  |
| *MSS11* | 13 | 587559 | T | G |  |  |  |
| *MSS11* | 13 | 587939 | A | G | Ser | Pro | 537 |
| *MSS11* | 13 | 587969 | T | C | Thr | Ala | 527 |
| *MSS11* | 13 | 588065 | A | G | Tyr | His | 495 |
| *MSS11* | 13 | 588112 | C | T | Ser | Asn | 479 |
| *MSS11* | 13 | 588348 | G | A |  |  |  |
| *MSS11* | 13 | 588660 | C | T | Gly | Glu | 92 |
| *MSS11* | 13 | 589273 | C | T |  |  |  |
| *MSS11* | 13 | 589360 | A | C | Ile | Ser | 63 |
| *MSS11* | 13 | 589414 | A | G | Val | Ala | 45 |
| Near *MSS11* | 13 | 589588 | T | C |  |  |  |
| *END3* | 14 | 470301 | C | T | Asp | Asn | 267 |
| *END3* | 14 | 470330 | C | T | Ser | Asn | 257 |
| *END3* | 14 | 470844 | A | G |  |  |  |
| *IRA2* | 15 | 171150 | T | C |  |  |  |
| *IRA2* | 15 | 171213 | G | A |  |  |  |
| *IRA2* | 15 | 171288 | T | C |  |  |  |
| *IRA2* | 15 | 171306 | C | T |  |  |  |
| *IRA2* | 15 | 171511 | A | C | Asn | His | 147 |
| *IRA2* | 15 | 171515 | G | A | Ser | Asn | 148 |
| *IRA2* | 15 | 171671 | A | G | Asn | Ser | 200 |
| *IRA2* | 15 | 171973 | T | C | Tyr | His | 301 |
| *IRA2* | 15 | 171981 | C | T |  |  |  |
| *IRA2* | 15 | 171985 | C | G | His | Asp | 305 |
| *IRA2* | 15 | 172102 | G | A | Ala | Thr | 344 |
| *IRA2* | 15 | 172468 | A | G | Asn | Asp | 466 |
| *IRA2* | 15 | 172515 | T | C |  |  |  |
| *IRA2* | 15 | 172581 | T | C |  |  |  |
| *IRA2* | 15 | 172704 | C | A |  |  |  |
| *IRA2* | 15 | 172752 | C | T |  |  |  |
| *IRA2* | 15 | 172768 | G | A | Val | Ile | 566 |
| *IRA2* | 15 | 172825 | C | T |  |  |  |
| *IRA2* | 15 | 172956 | A | G |  |  |  |
| *IRA2* | 15 | 173022 | A | G |  |  |  |
| *IRA2* | 15 | 173080 | G | A | Val | Met | 670 |
| *IRA2* | 15 | 173105 | A | T | Asn | Ile | 678 |
| *IRA2* | 15 | 173112 | T | C |  |  |  |
| *IRA2* | 15 | 173127 | T | C |  |  |  |
| *IRA2* | 15 | 173169 | T | C |  |  |  |
| *IRA2* | 15 | 173202 | A | G |  |  |  |
| *IRA2* | 15 | 173340 | A | C | Gln | His | 756 |
| *IRA2* | 15 | 173391 | A | G |  |  |  |
| *IRA2* | 15 | 173814 | T | C |  |  |  |
| *IRA2* | 15 | 173829 | C | T |  |  |  |
| *IRA2* | 15 | 174019 | A | T | Ser | Cys | 983 |
| *IRA2* | 15 | 174201 | A | G |  |  |  |
| *IRA2* | 15 | 174364 | C | T | Pro | Ser | 1098 |
| *IRA2* | 15 | 174465 | G | T |  |  |  |
| *IRA2* | 15 | 174472 | C | T | Leu | Phe | 1134 |
| *IRA2* | 15 | 174675 | T | C |  |  |  |
| *IRA2* | 15 | 174678 | T | C |  |  |  |
| *IRA2* | 15 | 175135 | A | G | Ile | Val | 1355 |
| *IRA2* | 15 | 175142 | C | T | Ser | Phe | 1357 |
| *IRA2* | 15 | 175290 | T | C |  |  |  |
| *IRA2* | 15 | 175332 | A | G |  |  |  |
| *IRA2* | 15 | 175458 | A | C |  |  |  |
| *IRA2* | 15 | 175470 | T | C |  |  |  |
| *IRA2* | 15 | 175512 | C | T |  |  |  |
| *IRA2* | 15 | 175815 | G | C |  |  |  |
| *IRA2* | 15 | 176091 | G | A |  |  |  |
| *IRA2* | 15 | 176217 | G | A |  |  |  |
| *IRA2* | 15 | 176239 | G | T | Ala | Ser | 1723 |
| *IRA2* | 15 | 176268 | A | T | Lys | Asn | 1732 |
| *IRA2* | 15 | 176298 | A | G |  |  |  |
| *IRA2* | 15 | 176425 | C | T |  |  |  |
| *IRA2* | 15 | 176441 | C | T | Ser | Leu | 1790 |
| *IRA2* | 15 | 176466 | C | T |  |  |  |
| *IRA2* | 15 | 176469 | C | T |  |  |  |
| *IRA2* | 15 | 177015 | A | G |  |  |  |
| *IRA2* | 15 | 177067 | T | G | Phe | Val | 1999 |
| *IRA2* | 15 | 177336 | C | T |  |  |  |
| *IRA2* | 15 | 177351 | T | C |  |  |  |
| *IRA2* | 15 | 177495 | A | G |  |  |  |
| *IRA2* | 15 | 177738 | G | A |  |  |  |
| *IRA2* | 15 | 177798 | T | C |  |  |  |
| *IRA2* | 15 | 178021 | C | T | Pro | Ser | 2363 |
| *IRA2* | 15 | 178159 | C | T |  |  |  |
| *IRA2* | 15 | 178287 | A | G |  |  |  |
| *IRA2* | 15 | 178296 | C | T |  |  |  |
| *IRA2* | 15 | 178440 | T | C |  |  |  |
| *IRA2* | 15 | 178474 | T | C |  |  |  |
| *IRA2* | 15 | 178587 | G | A |  |  |  |
| *IRA2* | 15 | 179031 | T | C |  |  |  |
| *IRA2* | 15 | 179291 | C | T | Ala | Val | 2740 |
| *IRA2* | 15 | 179481 | C | T |  |  |  |
| *IRA2* | 15 | 179652 | T | G | Phe | Leu | 2860 |
| *IRA2* | 15 | 179850 | A | G |  |  |  |
| *IRA2* | 15 | 180056 | A | C | Asn | Thr | 2995 |
| *IRA2* | 15 | 180123 | G | C |  |  |  |
| *IRA2* | 15 | 180159 | A | G |  |  |  |
| *IRA2* | 15 | 180177 | G | A |  |  |  |
| *IRA2* | 15 | 180210 | C | T |  |  |  |
