# Supplementary material for: Genetic Interactions Involving Five or More Genes Contribute to a Complex Trait in Yeast
Source: PLoS Genet. 2014 May 1;10(5):e1004324. doi: 10.1371/journal.pgen.1004324 (PMC4006734; doi:10.1371/journal.pgen.1004324)
Supplement: Table S8 — Genotypes within each phenotypic class among tetrad spores from the backcross to BY. (DOCX) [file pgen.1004324.s014.docx]

| genotype | rough | smooth |
| --- | --- | --- |
| *TRRI^3S^*/*FLO8^3S^*/*IRA2^3S^* | 7 | 4 |
| *TRRI^BY^*/*FLO8^3S^*/*IRA2^3S^* | 0 | 5 |
| *TRRI^3S^*/*FLO8^BY^*/*IRA2^3S^* | 0 | 3 |
| *TRRI^3S^*/*FLO8^3S^*/*IRA2^BY^* | 0 | 8 |
| *TRRI^BY^*/*FLO8^BY^*/*IRA2^3S^* | 0 | 9 |
| *TRRI^BY^*/*FLO8^3S^*/*IRA2^BY^* | 0 | 4 |
| *TRRI^3S^*/*FLO8^BY^*/*IRA2^BY^* | 0 | 6 |
| *TRRI^BY^*/*FLO8^BY^*/*IRA2^BY^* | 0 | 10 |
