# Supplementary material for: Genetic Interactions Involving Five or More Genes Contribute to a Complex Trait in Yeast
Source: PLoS Genet. 2014 May 1;10(5):e1004324. doi: 10.1371/journal.pgen.1004324 (PMC4006734; doi:10.1371/journal.pgen.1004324)
Supplement: Table S9 — Genotypes within each phenotypic class among tetrad spores from the backcross to 3S. (DOCX) [file pgen.1004324.s015.docx]

| genotype | rough | smooth |
| --- | --- | --- |
| *MSS11^BY^*/*END3^BY^* | 9 | 5 |
| *MSS11I^3S^*/*END3^BY^* | 0 | 11 |
| *MSS11^BY^*/*END3^3S^* | 2 | 10 |
| *MSS11^3S^*/*END3^3S^* | 0 | 15 |
