# Supplementary material for: Genetic Interactions Involving Five or More Genes Contribute to a Complex Trait in Yeast
Source: PLoS Genet. 2014 May 1;10(5):e1004324. doi: 10.1371/journal.pgen.1004324 (PMC4006734; doi:10.1371/journal.pgen.1004324)
Supplement: Table S10 — Bounds of fixed loci among rough individuals with END33S. Four sequenced segregants with rough morphology possessed END33S. These individuals shared 11 previously undetected loci. Intervals we later identify as causal are in bold. (DOCX) [file pgen.1004324.s016.docx]

| chromosome | start | stop |
| --- | --- | --- |
| I | 114628 | 227733 |
| II | 1687 | 19967 |
| V | 177250 | 254385 |
| **VII** | **949001** | **1009506** |
| VIII | 380512 | 489755 |
| XI | 85423 | 155453 |
| XI | 578824 | 625172 |
| XII | 210695 | 397605 |
| XIII | 823029 | 864754 |
| XV | 138746 | 143022 |
| **XV** | **586701** | **736524** |
