# Supplementary material for: Genetic Interactions Involving Five or More Genes Contribute to a Complex Trait in Yeast
Source: PLoS Genet. 2014 May 1;10(5):e1004324. doi: 10.1371/journal.pgen.1004324 (PMC4006734; doi:10.1371/journal.pgen.1004324)
Supplement: Table S11 — Genotyping of additional rough individuals possessing END33S. Two rough individuals with END33S were typed across loci identified in Figure S6. A 1 indicates that all genotyped individuals possessed the BY allele at a given marker and 0 indicates the 3S allele. (DOCX) [file pgen.1004324.s017.docx]

|  | typed individual | |
| --- | --- | --- |
| typed loci | A1 | A2 |
| chrI | 0 | 0 |
| chrII | 1 | 0 |
| chrV | 1 | 0 |
| chrVII | 1 | 1 |
| chrVIII | 0 | 1 |
| chrXI-1 | 1 | 1 |
| chrXI-2 | 0 | 1 |
| chrXII | 1 | 1 |
| chrXIII | 0 | 0 |
| chrXV-1 | 1 | 0 |
| chrXV-2 | 1 | 1 |
