# Supplementary material for: Genetic Interactions Involving Five or More Genes Contribute to a Complex Trait in Yeast
Source: PLoS Genet. 2014 May 1;10(5):e1004324. doi: 10.1371/journal.pgen.1004324 (PMC4006734; doi:10.1371/journal.pgen.1004324)
Supplement: Table S12 — Genotyping of rough END33S×3S second-generation backcross segregants. 39 individuals with rough morphology (1–39) and 12 with smooth morphology (c1–c12) were genotyped at segregating markers within four candidate loci. A 1 indicates that all genotyped individuals possessed the 3S allele at a given marker and 0 indicates the BY allele. (DOCX) [file pgen.1004324.s018.docx]

| individual | chrVII | chrXI | chrXII | chrXV |
| --- | --- | --- | --- | --- |
| 1 | 1 | 1 | 0 | 1 |
| 2 | 1 | 1 | 1 | 1 |
| 3 | 1 | 0 | 0 | 1 |
| 4 | 1 | 0 | 0 | 1 |
| 5 | 1 | 1 | 1 | 1 |
| 6 | 1 | 0 | 1 | 1 |
| 7 | 1 | 0 | 1 | 1 |
| 8 | 1 | 1 | 0 | 1 |
| 9 | 1 | 0 | 0 | 1 |
| 10 | 1 | 1 | 1 | 1 |
| 11 | 1 | 0 | 1 | 1 |
| 12 | 1 | 1 | 0 | 1 |
| 13 | 1 | 0 | 0 | 1 |
| 14 | 1 | 0 | 0 | 1 |
| 15 | 1 | 1 | 1 | 1 |
| 16 | 1 | 1 | 0 | 1 |
| 17 | 1 | 1 | 1 | 1 |
| 18 | 1 | 1 | 0 | 1 |
| 19 | 1 | 0 | 1 | 1 |
| 20 | 1 | 0 | 0 | 1 |
| 21 | 1 | 0 | 1 | 1 |
| 22 | 1 | 1 | 0 | 1 |
| 23 | 1 | 0 | 0 | 1 |
| 24 | 1 | 1 | 1 | 1 |
| 25 | 1 | 0 | 0 | 1 |
| 26 | 1 | 1 | 1 | 1 |
| 27 | 1 | 0 | 1 | 1 |
| 28 | 1 | 1 | 0 | 1 |
| 29 | 1 | 0 | 1 | 1 |
| 30 | 1 | 0 | 0 | 1 |
| 31 | 1 | 1 | 0 | 1 |
| 32 | 1 | 0 | 1 | 1 |
| 33 | 1 | 0 | 1 | 1 |
| 34 | 1 | 1 | 0 | 1 |
| 35 | 1 | 1 | 1 | 1 |
| 36 | 1 | 0 | 0 | 1 |
| 37 | 1 | 0 | 0 | 1 |
| 38 | 1 | 0 | 0 | 1 |
| 39 | 1 | 1 | 1 | 1 |
| c1 | 0 | 0 | 1 | 0 |
| c2 | 0 | 1 | 1 | 0 |
| c3 | 1 | 0 | 0 | 0 |
| c4 | 1 | 0 | 0 | 1 |
| c5 | 0 | 1 | 1 | 0 |
| c6 | 1 |  | 0 | 1 |
| c7 | 1 | 0 | 1 | 1 |
| c8 | 0 | 1 | 0 | 0 |
| c9 | 1 | 1 | 0 | 1 |
| c10 | 1 | 0 | 1 | 1 |
| c11 | 1 | 1 | 0 | 0 |
| c12 | 1 | 1 | 1 | 1 |
