# Supplementary material for: Genetic Interactions Involving Five or More Genes Contribute to a Complex Trait in Yeast
Source: PLoS Genet. 2014 May 1;10(5):e1004324. doi: 10.1371/journal.pgen.1004324 (PMC4006734; doi:10.1371/journal.pgen.1004324)
Supplement: Table S13 — Genes within additional loci tested by deletion. Each of the above genes were tested for involvement in rough morphology by deletion in a rough individual. (DOCX) [file pgen.1004324.s019.docx]

| gene | chromosome | position |
| --- | --- | --- |
| *PHO81* | VII | 958210 to 954674 |
| *YAP1802* | VII | 976581 to 974875 |
| *SDA1* | VII | 982068 to 979765 |
| *SOL4* | VII | 985972 to 986739 |
| *MGA1* | VII | 988049 to 989419 |
| *SFL1* | XV | 586981 to 589281 |
| *ELG1* | XV | 605092 to 602717 |
| *PNO1* | XV | 606171 to 605347 |
| *MDM32* | XV | 606607 to 608475 |
| *SPP2* | XV | 609197 to 608640 |
| *SMP3* | XV | 611388 to 609838 |
| *MRPL23* | XV | 611999 to 612490 |
| *RPB2* | XV | 616671 to 612997 |
| *YOR152C* | XV | 618288 to 617518 |
| *PDR5* | XV | 619840 to 624375 |
| *SLP1* | XV | 624729 to 626492 |
| *LAS17* | XV | 675939 to 677840 |
| *BFR1* | XV | 720065 to 718653 |
