# Supplementary material for: Genetic Interactions Involving Five or More Genes Contribute to a Complex Trait in Yeast
Source: PLoS Genet. 2014 May 1;10(5):e1004324. doi: 10.1371/journal.pgen.1004324 (PMC4006734; doi:10.1371/journal.pgen.1004324)
Supplement: Text S1 — Evaluation of the essential genes AVO1 and TOP2. (DOCX) [file pgen.1004324.s020.docx]

Because *AVO1* and *TOP2* are essential for viability, we could not delete these genes from a rough haploid individual. We used an alternate approach to test the effects of these genes. Heterozygous diploids for the loci containing these genes were generated by mating a rough multi-locus introgression strain to an appropriate backcross segregant. Deletion of *AVO1* and *TOP2* in these diploid heterozygotes generated mixed populations of hemizygotes, with approximately equal numbers of individuals carrying the BY and 3S alleles of these genes. All hemizygotes exhibited rough morphology, suggesting that these genes do not harbor functional variation that affects the trait.
