# Supplementary material for: Genetic Interactions Involving Five or More Genes Contribute to a Complex Trait in Yeast
Source: PLoS Genet. 2014 May 1;10(5):e1004324. doi: 10.1371/journal.pgen.1004324 (PMC4006734; doi:10.1371/journal.pgen.1004324)
Supplement: Text S2 — Protein sequences of Ira2BY, Ira23S, and Ira23S -Δ2933. (DOCX) [file pgen.1004324.s021.docx]

1 10 20 30 40 50 60

| | | | | | |

IRA23S-Δ2933 MSQPTKNKKKEHGTDSKSSRMTRTLVNHILFERILPILPVESNLSTYSEVEEYSSFISCR

IRA23S MSQPTKNKKKEHGTDSKSSRMTRTLVNHILFERILPILPVESNLSTYSEVEEYSSFISCR

IRA2BY MSQPTKNKKKEHGTDSKSSRMTRTLVNHILFERILPILPVESNLSTYSEVEEYSSFISCR

IRA23S-Δ2933 SVLINVTVSRDANAMVEGTLELIESLLQGHEIISDKGSSDVIESILIILRLLSDALEYNW

IRA23S SVLINVTVSRDANAMVEGTLELIESLLQGHEIISDKGSSDVIESILIILRLLSDALEYNW

IRA2BY SVLINVTVSRDANAMVEGTLELIESLLQGHEIISDKGSSDVIESILIILRLLSDALEYNW

IRA23S-Δ2933 QNQESLHYNDISTHVEHDQEQKYRPKLHNILPDYSSTHSNGNKHFFHQSKPQALIPELAS

IRA23S QNQESLHYNDISTHVEHDQEQKYRPKLHNILPDYSSTHSNGNKHFFHQSKPQALIPELAS

IRA2BY QNQESLHYNDISTHVEHDQEQKYRPKLNSILPDYSSTHSNGNKHFFHQSKPQALIPELAS

IRA23S-Δ2933 KLLESCAKLKFNTRTLQILQSMISHVHGNILTTLSSSILPRHKSYLTRHNHPSHCKMIDS

IRA23S KLLESCAKLKFNTRTLQILQSMISHVHGNILTTLSSSILPRHKSYLTRHNHPSHCKMIDS

IRA2BY KLLESCAKLKFNTRTLQILQNMISHVHGNILTTLSSSILPRHKSYLTRHNHPSHCKMIDS

IRA23S-Δ2933 TLGHILRFVAASNPSEYFEFIRKSVQVPVTQTHTHSHSHSHSLPSSVYNSIVPHFDLFSF

IRA23S TLGHILRFVAASNPSEYFEFIRKSVQVPVTQTHTHSHSHSHSLPSSVYNSIVPHFDLFSF

IRA2BY TLGHILRFVAASNPSEYFEFIRKSVQVPVTQTHTHSHSHSHSLPSSVYNSIVPHFDLFSF

IRA23S-Δ2933 IHLSKDNFKKYLELIKNLSVTLRKTIYHCLLLHYSAKAIMFWIMTRPAEYYELFNLLKDN

IRA23S IHLSKDNFKKYLELIKNLSVTLRKTIYHCLLLHYSAKAIMFWIMTRPAEYYELFNLLKDN

IRA2BY IYLSKHNFKKYLELIKNLSVTLRKTIYHCLLLHYSAKAIMFWIMARPAEYYELFNLLKDN

IRA23S-Δ2933 NNEHSKSLNTLNHTLFEEIHSTFNVNSMITTNQNAHQGSSSPSSSSPSSPPSSSSSDNNN

IRA23S NNEHSKSLNTLNHTLFEEIHSTFNVNSMITTNQNAHQGSSSPSSSSPSSPPSSSSSDNNN

IRA2BY NNEHSKSLNTLNHTLFEEIHSTFNVNSMITTNQNAHQGSSSPSSSSPSSPPSSSSSDNNN

IRA23S-Δ2933 QNIIAKSLSRQLSHHQSYIQQQSERKLHSSWTTNSQSSTSLSSSTSDSTTTDFSTHTQPG

IRA23S QNIIAKSLSRQLSHHQSYIQQQSERKLHSSWTTNSQSSTSLSSSTSDSTTTDFSTHTQPG

IRA2BY QNIIAKSLSRQLSHHQSYIQQQSERKLHSSWTTNSQSSTSLSSSTSNSTTTDFSTHTQPG

IRA23S-Δ2933 EYDPSLPDTPTMSNITISASSLLSQTPTPTTQLQQRLNSAAAAAAAAASPSNSTPTGYTA

IRA23S EYDPSLPDTPTMSNITISASSLLSQTPTPTTQLQQRLNSAAAAAAAAASPSNSTPTGYTA

IRA2BY EYDPSLPDTPTMSNITISASSLLSQTPTPTTQLQQRLNSAAAAAAAAASPSNSTPTGYTA

IRA23S-Δ2933 EQQSRASYDAHKTGHTGKDYDEHFLSITRLDNVLELYTHFDDTEVLPHTSVLKFLTTLTM

IRA23S EQQSRASYDAHKTGHTGKDYDEHFLSITRLDNVLELYTHFDDTEVLPHTSVLKFLTTLTM

IRA2BY EQQSRASYDAHKTGHTGKDYDEHFLSVTRLDNVLELYTHFDDTEVLPHTSVLKFLTTLTM

IRA23S-Δ2933 FDIDLFNELNATSFKYIPDCTMHRPKERTSSFNNTAHETGSEKTSGIKHITQGLKKLTSL

IRA23S FDIDLFNELNATSFKYIPDCTMHRPKERTSSFNNTAHETGSEKTSGIKHITQGLKKLTSL

IRA2BY FDIDLFNELNATSFKYIPDCTMHRPKERTSSFNNTAHETGSEKTSGIKHITQGLKKLTSL

IRA23S-Δ2933 PSSTKKTVKFMKMLLRNLIGNQAVSDVALLDTMRALLSFFTMTSAVFLVDRNLPSVLFAK

IRA23S PSSTKKTVKFMKMLLRNLIGNQAVSDVALLDTMRALLSFFTMTSAVFLVDRNLPSVLFAK

IRA2BY PSSTKKTVKFVKMLLRNLNGNQAVSDVALLDTMRALLSFFTMTSAVFLVDRNLPSVLFAK

IRA23S-Δ2933 RLIPIMGTNLSVGQDWNSKINNSLMVCLKKNSTTFVHLQLIFFSSAIQFDHELLLARLSI

IRA23S RLIPIMGTNLSVGQDWNSKINNSLMVCLKKNSTTFVHLQLIFFSSAIQFDHELLLARLSI

IRA2BY RLIPIMGTNLSVGQDWNSKINNSLMVCLKKNSTTFVQLQLIFFSSAIQFDHELLLARLSI

IRA23S-Δ2933 DTMANNLNMQKLCLYTEGFRIFFDIPSKKELRKAIAVKISKFFKTLFSIIADILLQEFPY

IRA23S DTMANNLNMQKLCLYTEGFRIFFDIPSKKELRKAIAVKISKFFKTLFSIIADILLQEFPY

IRA2BY DTMANNLNMQKLCLYTEGFRIFFDIPSKKELRKAIAVKISKFFKTLFSIIADILLQEFPY

IRA23S-Δ2933 FDEQITDIVASILDGTIINEYGTKKHFKGSSPSLCSTTRSRSGSTSQSSMTPVSPLGLDT

IRA23S FDEQITDIVASILDGTIINEYGTKKHFKGSSPSLCSTTRSRSGSTSQSSMTPVSPLGLDT

IRA2BY FDEQITDIVASILDGTIINEYGTKKHFKGSSPSLCSTTRSRSGSTSQSSMTPVSPLGLDT

IRA23S-Δ2933 DICPMNTLSLVGSSTSRNSDNVNSLNSSPKNLSSDPYLSHLVAPRARHALGGPSSIIRNK

IRA23S DICPMNTLSLVGSSTSRNSDNVNSLNSSPKNLSSDPYLSHLVAPRARHALGGPSSIIRNK

IRA2BY DICPMNTLSLVGSSTSRNSDNVNSLNSSPKNLSSDPYLSHLVAPRARHALGGPSSIIRNK

IRA23S-Δ2933 IPTTLTSPPGTEKSSPVQRPQTECISATPMAITNSTPLSSAAFGIRSPLQKIRTRRYSDE

IRA23S IPTTLTSPPGTEKSSPVQRPQTECISATPMAITNSTPLSSAAFGIRSPLQKIRTRRYSDE

IRA2BY IPTTLTSPPGTEKSSPVQRPQTESISATPMAITNSTPLSSAAFGIRSPLQKIRTRRYSDE

IRA23S-Δ2933 SLGKFMKSTNNYIQEHLIPKDLNEATLQDARRIMINIFSIFKRPNSYFIIPHNINSNLQW

IRA23S SLGKFMKSTNNYIQEHLIPKDLNEATLQDARRIMINIFSIFKRPNSYFIIPHNINSNLQW

IRA2BY SLGKFMKSTNNYIQEHLIPKDLNEATLQDARRIMINIFSIFKRPNSYFIIPHNINSNLQW

IRA23S-Δ2933 VSQDFRNIMKPIFVAIVSSDVDLQNTAQSFMDTLLSNVITYGESDENISIEGYHFLCSYT

IRA23S VSQDFRNIMKPIFVAIVSSDVDLQNTAQSFMDTLLSNVITYGESDENISIEGYHFLCSYT

IRA2BY VSQDFRNIMKPIFVAIVSPDVDLQNTAQSFMDTLLSNVITYGESDENISIEGYHLLCSYT

IRA23S-Δ2933 VTLFAMGLFDLKINNEKRQILLDITVKFMKVRSHLAGIAEASHHMEYISDSEKLTFPLIM

IRA23S VTLFAMGLFDLKINNEKRQILLDITVKFMKVRSHLAGIAEASHHMEYISDSEKLTFPLIM

IRA2BY VTLFAMGLFDLKINNEKRQILLDITVKFMKVRSHLAGIAEASHHMEYISDSEKLTFPLIM

IRA23S-Δ2933 GTVGRALFVSLYSSQQKIEKTLKIAYTEYLSAINFHERNIDDADKTWVHNIEFVEAMCHD

IRA23S GTVGRALFVSLYSSQQKIEKTLKIAYTEYLSAINFHERNIDDADKTWVHNIEFVEAMCHD

IRA2BY GTVGRALFVSLYSSQQKIEKTLKIAYTEYLSAINFHERNIDDADKTWVHNIEFVEAMCHD

IRA23S-Δ2933 NYTTSGSIAFQRRTRNNILRFATIPNAILLDSMRMIYKKWHTYTHSKSLEKQERNDFRNF

IRA23S NYTTSGSIAFQRRTRNNILRFATIPNAILLDSMRMIYKKWHTYTHSKSLEKQERNDFRNF

IRA2BY NYTTSGSIAFQRRTRNNILRFATIPNAILLDSMRMIYKKWHTYTHSKSLEKQERNDFRNF

IRA23S-Δ2933 AGILASLSGILFINKKILQEMYPYLLDTVSELKKNVDFFISKQCQWLNYPDLLTRENSRD

IRA23S AGILASLSGILFINKKILQEMYPYLLDTVSELKKNVDFFISKQCQWLNYPDLLTRENSRD

IRA2BY AGILASLSGILFINKKILQEMYPYLLDTVSELKKNIDSFISKQCQWLNYPDLLTRENSRD

IRA23S-Δ2933 ILSVELHPLSFNLLFNNLRLKLKELACSDLSIPENESSYVLLEQIIKMLRTILGRDDDNY

IRA23S ILSVELHPLSFNLLFNNLRLKLKELACSDLSIPENESSYVLLEQIIKMLRTILGRDDDNY

IRA2BY ILSVELHPLSFNLLFNNLRLKLKELACSDLSIPENESSYVLLEQIIKMLRTILGRDDDNY

IRA23S-Δ2933 VMMLFSTEIVDLIDLLTDEIKKIPAYCPKYLKAIIQMTKMFSALQHSEVNLGVKNHFHVK

IRA23S VMMLFSTEIVDLIDLLTDEIKKIPAYCPKYLKAIIQMTKMFSALQHSEVNLGVKNHFHVK

IRA2BY VMMLFSTEIVDLIDLLTDEIKKIPAYCPKYLKAIIQMTKMFSALQHSEVNLGVKNHFHVK

IRA23S-Δ2933 NKWLRQITDWFQVSIAREYDFENLSKPLKEMDLVKRDMDILYIDTAIEASTAIAYLTRHT

IRA23S NKWLRQITDWFQVSIAREYDFENLSKPLKEMDLVKRDMDILYIDTAIEASTAIAYLTRHT

IRA2BY NKWLRQITDWFQVSIAREYDFENLSKPLKEMDLVKRDMDILYIDTAIEASTAIAYLTRHT

IRA23S-Δ2933 FLEIPPAASDPELSRSRSVIFGFYFNILMKGLEKSSDRDNYPVFLRHKMSVLNDNVILSL

IRA23S FLEIPPAASDPELSRSRSVIFGFYFNILMKGLEKSSDRDNYPVFLRHKMSVLNDNVILSL

IRA2BY FLEIPPAASDPELSRSRSVIFGFYFNILMKGLEKSSDRDNYPVFLRHKMSVLNDNVILSL

IRA23S-Δ2933 TNLSNTNVDASLQFTLPMGYSGNRNIRNAFLEVFINIVTNYRTYTAKTDLGKLEAADKFL

IRA23S TNLSNTNVDASLQFTLPMGYSGNRNIRNAFLEVFINIVTNYRTYTAKTDLGKLEAADKFL

IRA2BY TNLSNTNVDASLQFTLPMGYSGNRNIRNAFLEVFINIVTNYRTYTAKTDLGKLEAADKFL

IRA23S-Δ2933 RYTIEHPQLSSFGAAVCPASDIDAYAAGLINAFETRNATHIVVSQLIKNEIENSSRPTDI

IRA23S RYTIEHPQLSSFGAAVCPASDIDAYAAGLINAFETRNATHIVVSQLIKNEIENSSRPTDI

IRA2BY RYTIEHPQLSSFGAAVCPASDIDAYAAGLINAFETRNATHIVVAQLIKNEIEKSSRPTDI

IRA23S-Δ2933 LRRNSCATRSLSMLARSKGNEYLIRTLQPLLKKIIQNRDFFEIEKLKPEDLDAERQIELF

IRA23S LRRNSCATRSLSMLARSKGNEYLIRTLQPLLKKIIQNRDFFEIEKLKPEDLDAERQIELF

IRA2BY LRRNSCATRSLSMLARSKGNEYLIRTLQPLLKKIIQNRDFFEIEKLKPEDSDAERQIELF

IRA23S-Δ2933 VKYMNELLESISNSVSYFPPPLFYICQNIYKVACEKFPDHAIIAAGSFVFLRFFCPALVS

IRA23S VKYMNELLESISNSVSYFPPPLFYICQNIYKVACEKFPDHAIIAAGSFVFLRFFCPALVS

IRA2BY VKYMNELLESISNSVSYFPPPLFYICQNIYKVACEKFPDHAIIAAGSFVFLRFFCPALVS

IRA23S-Δ2933 PDSENIIDISHLSEKRTFISLAKVIQNIANGSENFSRWPALCSQKDFLKECSDRIFRFLA

IRA23S PDSENIIDISHLSEKRTFISLAKVIQNIANGSENFSRWPALCSQKDFLKECSDRIFRFLA

IRA2BY PDSENIIDISHLSEKRTFISLAKVIQNIANGSENFSRWPALCSQKDFLKECSDRIFRFLA

IRA23S-Δ2933 ELCRTDRTIDIQVRTDPTPIAFDYQFLHSFVYLYGLEVRRNVLNEAKHDDGDIDGDDFYK

IRA23S ELCRTDRTIDIQVRTDPTPIAFDYQFLHSFVYLYGLEVRRNVLNEAKHDDGDIDGDDFYK

IRA2BY ELCRTDRTIDIQVRTDPTPIAFDYQFLHSFVYLYGLEVRRNVLNEAKHDDGDIDGDDFYK

IRA23S-Δ2933 TTFLLIDDVLGQLGQPKMEVSNEIPIYIREHMDDYPELYEFMNRHAFRNIETSTAYSPSV

IRA23S TTFLLIDDVLGQLGQPKMEVSNEIPIYIREHMDDYPELYEFMNRHAFRNIETSTAYSPSV

IRA2BY TTFLLIDDVLGQLGQPKMEFSNEIPIYIREHMDDYPELYEFMNRHAFRNIETSTAYSPSV

IRA23S-Δ2933 HESTSSEGIPIITLTMSNFSDRHVDIDTVAYKFLQIYARIWTTKHCLIIDCTEFDEGGLD

IRA23S HESTSSEGIPIITLTMSNFSDRHVDIDTVAYKFLQIYARIWTTKHCLIIDCTEFDEGGLD

IRA2BY HESTSSEGIPIITLTMSNFSDRHVDIDTVAYKFLQIYARIWTTKHCLIIDCTEFDEGGLD

IRA23S-Δ2933 MRKFISLVMGLLPEVAPKNCIGCYYFNVNETFMDNYGKCLDKDNVYVSSKIPHYFINSNS

IRA23S MRKFISLVMGLLPEVAPKNCIGCYYFNVNETFMDNYGKCLDKDNVYVSSKIPHYFINSNS

IRA2BY MRKFISLVMGLLPEVAPKNCIGCYYFNVNETFMDNYGKCLDKDNVYVSSKIPHYFINSNS

IRA23S-Δ2933 DEGLMKSVGITGQGLKVLQDIRVSLHDITLYDEKRNRFTPVSLKIGDIYFQVLHETPRQY

IRA23S DEGLMKSVGITGQGLKVLQDIRVSLHDITLYDEKRNRFTPVSLKIGDIYFQVLHETPRQY

IRA2BY DEGLMKSVGITGQGLKVLQDIRVSLHDITLYDEKRNRFTPVSLKIGDIYFQVLHETPRQY

IRA23S-Δ2933 KIRDMGTLFDVKFNDVYEISRIFEVHVSSITGVAAEFTVTFQDERRLIFSSPKYLEIVKM

IRA23S KIRDMGTLFDVKFNDVYEISRIFEVHVSSITGVAAEFTVTFQDERRLIFSSPKYLEIVKM

IRA2BY KIRDMGTLFDVKFNDVYEISRIFEVHVSSITGVAAEFTVTFQDERRLIFSSPKYLEIVKM

IRA23S-Δ2933 FYYAQIRLESEYEMDNNSSTSSPNSNNKDKQQKERTKLLCHLLLVSLIGLFDESKKMKNS

IRA23S FYYAQIRLESEYEMDNNSSTSSPNSNNKDKQQKERTKLLCHLLLVSLIGLFDESKKMKNS

IRA2BY FYYAQIRLESEYEMDNNSSTSSPNSNNKDKQQKERTKLLCHLLLVSLIGLFDESKKMKNS

IRA23S-Δ2933 SYNLIAATEASFGLNFGSHFHRSSEVYVPEDTTTFLGVIGKSLAESNPELTAYMFIYVLE

IRA23S SYNLIAATEASFGLNFGSHFHRSSEVYVPEDTTTFLGVIGKSLAESNPELTAYMFIYVLE

IRA2BY SYNLIAATEASFGLNFGSHFHRSPEVYVPEDTTTFLGVIGKSLAESNPELTAYMFIYVLE

IRA23S-Δ2933 ALKNNVIPHVYIPHTICGLSYWIPNLYQHVYLADDEEGPENISHIFRILIRLSVRETDFK

IRA23S ALKNNVIPHVYIPHTICGLSYWIPNLYQHVYLADDEEGPENISHIFRILIRLSVRETDFK

IRA2BY ALKNNVIPHVYIPHTICGLSYWIPNLYQHVYLADDEEGPENISHIFRILIRLSVRETDFK

IRA23S-Δ2933 AVYMQYVWLLLLDDGRLTDIIVDEVINHALERDSENRDWKKTISLLTVLPTTEVANNIIQ

IRA23S AVYMQYVWLLLLDDGRLTDIIVDEVINHALERDSENRDWKKTISLLTVLPTTEVANNIIQ

IRA2BY AVYMQYVWLLLLDDGRLTDIIVDEVINHALERDSENRDWKKTISLLTVLPTTEVANNIIQ

IRA23S-Δ2933 KILAKIRSFLPSLKLEAMTQSWSELTILVKISIHVFFETSLLVQMYLPEILFIVSLLIDV

IRA23S KILAKIRSFLPSLKLEAMTQSWSELTILVKISIHVFFETSLLVQMYLPEILFIVSLLIDV

IRA2BY KILAKIRSFLPSLKLEAMTQSWSELTILVKISIHVFFETSLLVQMYLPEILFIVSLLIDV

IRA23S-Δ2933 GPRELRSSLHQLLMNVCHSLAINSALPQDHRNNLDEISDIFAHQKVKFMFGFSEDKGRIL

IRA23S GPRELRSSLHQLLMNVCHSLAINSALPQDHRNNLDEISDIFAHQKVKFMFGFSEDKGRIL

IRA2BY GPRELRSSLHQLLMNVCHSLAINSALPQDHRNNLDEISDIFAHQKVKFMFGFSEDKGRIL

IRA23S-Δ2933 QIFSASSFASKFNILDFFINNILLLMEYSSTYEANVWKTRYKKYVLESVFTSNSFLSARS

IRA23S QIFSASSFASKFNILDFFINNILLLMEYSSTYEANVWKTRYKKYVLESVFTSNSFLSARS

IRA2BY QIFSASSFASKFNILDFFINNILLLMEYSSTYEANVWKTRYKKYVLESVFTSNSFLSARS

IRA23S-Δ2933 IMIVGIMGKSYITEGLCKAMLIETMKVIAEPKITDEHLFLVISHIFTYSKIVEGLDPNLD

IRA23S IMIVGIMGKSYITEGLCKAMLIETMKVIAEPKITDEHLFLVISHIFTYSKIVEGLDPNLD

IRA2BY IMIVGIMGKSYITEGLCKAMLIETMKVIAEPKITDEHLFLAISHIFTYSKIVEGLDPNLD

IRA23S-Δ2933 LMKHLFWFSTLFLESRHPIIFEGALLFVSNCIRRLYMAQFENESETSLISTLLKGRKFAH

IRA23S LMKHLFWFSTLFLESRHPIIFEGALLFVSNCIRRLYMAQFENESETSLISTLLKGRKFAH

IRA2BY LMKHLFWFSTLFLESRHPIIFEGALLFVSNCIRRLYMAQFENESETSLISTLLKGRKFAH

IRA23S-Δ2933 TFLSKIENLSGIVWNEDNFTHILIFIINKGLSNPFIKSTALDFLKMMFRNSYFEHQINQK

IRA23S TFLSKIENLSGIVWNEDNFTHILIFIINKGLSNPFIKSTALDFLKMMFRNSYFEHQINQK

IRA2BY TFLSKIENLSGIVWNEDNFTHILIFIINKGLSNPFIKSTAFDFLKMMFRNSYFEHQINQK

IRA23S-Δ2933 SDHYLCYMFLLYFVLNCNQFEELLGDVDFEGEMVNIENKNTIPKILLEWLSSDTKMQTLP

IRA23S SDHYLCYMFLLYFVLNCNQFEELLGDVDFEGEMVNIENKNTIPKILLEWLSSDNENANIT

IRA2BY SDHYLCYMFLLYFVLNCNQFEELLGDVDFEGEMVNIENKNTIPKILLEWLSSDNENANIT

IRA23S-Δ2933 SIKVRYCSNVQLRMNQVDLGLR--------------------------------------

IRA23S LYQGAILFKCSVTDEPSRFRFALIIRHLLTKKPICALRFYSVIRNEIRKISAFEQTSDCV

IRA2BY LYQGAILFKCSVTDEPSRFRFALIIRHLLTKKPICALRFYSVIRNEIRKISAFEQNSDCV

IRA23S-Δ2933 ------------------------------------------------------------

IRA23S PLAFDILNLLVTHSESNSLEKLHEESIERLTKRGLSIVTSSGIFAKNSDMMIPLDVKPED

IRA2BY PLAFDILNLLVTHSESNSLEKLHEESIERLTKRGLSIVTSSGIFAKNSDMMIPLDVKPED

IRA23S-Δ2933 -------------------

IRA23S IYERKRIMTMILSRMSCSA

IRA2BY IYERKRIMTMILSRMSCSA
